# Are *PECTIN ESTERASE INHIBITOR* Genes Involved in Mediating Resistance to *Rhynchosporium commune* in Barley?

**DOI:** 10.1371/journal.pone.0150485

**Published:** 2016-03-03

**Authors:** Stephan Marzin, Anja Hanemann, Shailendra Sharma, Götz Hensel, Jochen Kumlehn, Günther Schweizer, Marion S. Röder

**Affiliations:** 1 Leibniz Institute of Plant Genetics and Crop Plant Research, Gatersleben, Germany; 2 Bayerische Landesanstalt für Landwirtschaft, Freising, Germany; Julius Kuehn-Institute (JKI), GERMANY

## Abstract

A family of putative *PECTIN ESTERASE INHIBITOR* (*PEI*) genes, which were detected in the genomic region co-segregating with the resistance gene *Rrs2* against scald caused by *Rhynchosporium commune* in barley, were characterized and tested for their possible involvement in mediating resistance to the pathogen by complementation and overexpression analysis. The sequences of the respective genes were derived from two BAC contigs originating from the susceptible cultivar ‘Morex’. For the genes *HvPEI2*, *HvPEI3*, *HvPEI4* and *HvPEI6*, specific haplotypes for 18 resistant and 23 susceptible cultivars were detected after PCR-amplification and haplotype-specific CAPS-markers were developed. None of the tested candidate genes *HvPEI2*, *HvPEI3* and *HvPEI4* alone conferred a high resistance level in transgenic over-expression plants, though an improvement of the resistance level was observed especially with OE-lines for gene *HvPEI4*. These results do not confirm but also do not exclude an involvement of the *PEI* gene family in the response to the pathogen. A candidate for the resistance gene *Rrs2* could not be identified yet. It is possible that *Rrs2* is a *PEI* gene or another type of gene which has not been detected in the susceptible cultivar ‘Morex’ or the full resistance reaction requires the presence of several *PEI* genes.

## Introduction

Barley (*Hordeum vulgare* L.), one of the most important crop species worldwide is threatened by the hemibiotrophic, haploid ascomycete *Rhynchosporium commune (*formerly named *Rhynchosporium secalis* (Oudem.) J.J. Davis) [1–3], which causes a necrotic foliar disease (scald or leaf blotch) on infected plants [4]. Recent studies revealed that the genus *Rhynchosporium* which consists of two species *R*. *secalis* and *R*. *orthosporum* can be further divided based on phylogenetic criteria [5]. *R*. *secalis* separates into three monophyletic groups that are also characterized by their host spectra: *R*. *secalis* infecting rye and triticale, *R*. *commune* infecting cultivated barley and other *Hordeum* spp. as well as *Bromus diandrus* (brome grass) and *R*. *agropyri*, infecting Agropyron spp. [5].

Regarding the resistance of barley against *R*. *commune*, research has led to the identification of altogether 16 major resistance genes or alleles (*R* genes), however only nine of them have been mapped until now [6,7], which include four major resistance loci such as the *Rrs1* complex on chromosome 3H with at least 11 known alleles [8,9], the *Rrs2* locus on 7HS [10,11], *Rrs13* on chromosome 6H [12] and the *Rrs15* locus on 2H [13].

Our knowledge is still limited regarding the identity of the mapped resistance genes and their possible functions and resistance mechanisms. For the *Rrs1* mediated resistance, prevention of subcuticular hyphae growth has been observed to be a major factor in the defence reaction against *R*. *commune* [1]. Jørgensen et al. [14] found that the *Rrs2* mediated resistance reaction in the cultivars ‘Digger’ and ‘Osiris’, which both carry the *Rrs2* gene, is characterized by the prevention of fungal penetration of the cuticle through an accelerated and larger formation of papillae and halos in the cell walls compared to susceptible cultivars. The authors also suspect the involvement of chemical compounds in the inhibition process.

Pectins account for ca. 30% of the cell walls of dicot and non-graminaceous monocot plants and for between 5–10% of the walls of grasses. They are a family of complex polysaccharides whose backbones are highly enriched in α-1,4-linked-D-galacturonic acid residues, with various degrees of methylesterification [15]. Pectin is secreted in highly methyl-esterified form and subsequently deesterified by pectin methylesterases (*PMEs*; EC 3.1.1.11). PME activity is controlled by pectin methylesterase inhibitors (*PMEI* or *PEI*), which belong to the family of invertase inhibitors [15]. *PMEs* as well as *PMEIs* appear to occur as large gene families. In *Arabidopsis thaliana*, 66 ORFs have been annotated as putative full-length *PMEs* and 35 ORFs in *Oryza sativa* [16]. The structural details of *PME-PMEI* interaction have been studied by crystallographic methods in several plant species and the inhibition apparently occurs through the formation of an inactive 1:1 complex between the two proteins [17–20]. The role of PMEs in plants have been implicated in several processes, such as cell wall extension, fruit maturation, senescence, pollen tube growth and seed germination [15,21,22]. Furthermore, *PMEs* are involved in the systemic spread of tobacco mosaic virus [23] as well as tobamovirus [24] and seem to stimulate virus-induced gene silencing [25]. An involvement of cell walls and *PME* action in resistance to pathogens has been discussed [15,16,26–28].

Our previous results of fine mapping, in which we used a segregating population ‘Atlas’ (resistant) × ‘Steffi’ (susceptible) of 9,179 F_2_ plants, assigned the scald resistance gene *Rrs2* to an area of suppressed recombination on chromosome 7HS [29]. Based on these fine mapping results we decided to carry out a sequence survey of this suppressed recombination region in order to pinpoint interesting genes depicting haplotype variation between resistant and susceptible cultivars. Analysis of several genes, identified within the region of interest, led to the discovery of a family of putative *PECTIN ESTERASE INHIBITORs* (*PEI*) whose haplotypes differed between resistant and susceptible cultivars. This feature and the fact that these genes co-segregated with the resistance gene *Rrs2* made them possible candidates for the *Rrs2* gene. The goal of the present research work was to elucidate the role of the putative *PECTIN ESTERASE INHIBITOR* (*PEI*) genes, in the process of mediating resistance to *R*. *commune* in barley.

## Results

### The PEI-gene family in barley

**Fig 1:**
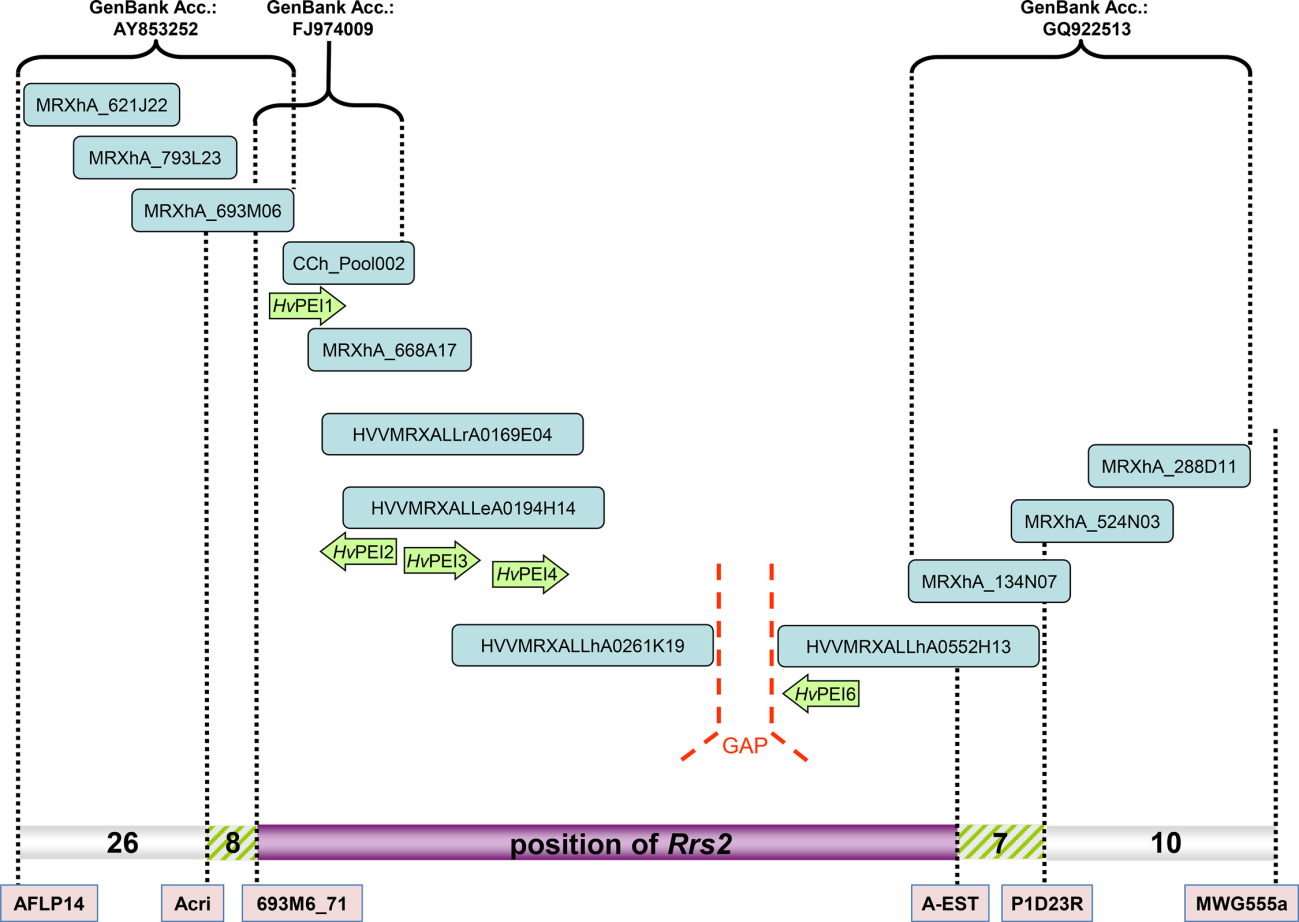
BAC contig of the *Rrs*2 region carrying various *PEI*-genes. The horizontal bar represents the genetic map based on 9,179 F_2_-plants with the numbers of recombinant plants between six genetic markers indicated according to [29]. The hatched areas represent the location of recombination events and could also harbor the *Rrs2*-gene. The blue boxes represent BAC-clones and the green arrows indicate the approximate locations of the PEI genes.

The *Rrs*2 gene which confers resistance to *R*. *commune* was fine mapped by Hanemann et al. [29]. It is located in a co-segregating genomic region on barley chromosome 7HS. In previous research we had established two BAC contigs for the co-segregating region, which consisted of BACs from the susceptible cultivar ‘Morex’ [29]. In this genomic region a number of genes had been identified including genes coding for putative pectinesterase inhibitor domain containing proteins [29]. Further analysis of BAC sequences that became available during the past two years [30] led to the discovery of additional *PEI* family members in the neighborhood of already known *PEI* candidates (Fig 1). Gene ontology description predicted enzyme inhibitor activity (GO:0004857) for pectin esterase activity (GO:0030599) for genes *HvPEI1*, *HvPEI2*, *HvPEI3*, *HvPEI4* and *HvPEI6*, therefore we refer to this gene family as putative pectin esterase inhibitor genes. For *HvPEI6* additionally an ABC transporter related InterPro description was identified with molecular functions of ATP binding (GO:0005524) and ATPase activity (GO:0016887), while for *HvPEI5* no gene ontology function was identified (S1 File).

**Fig 2:**
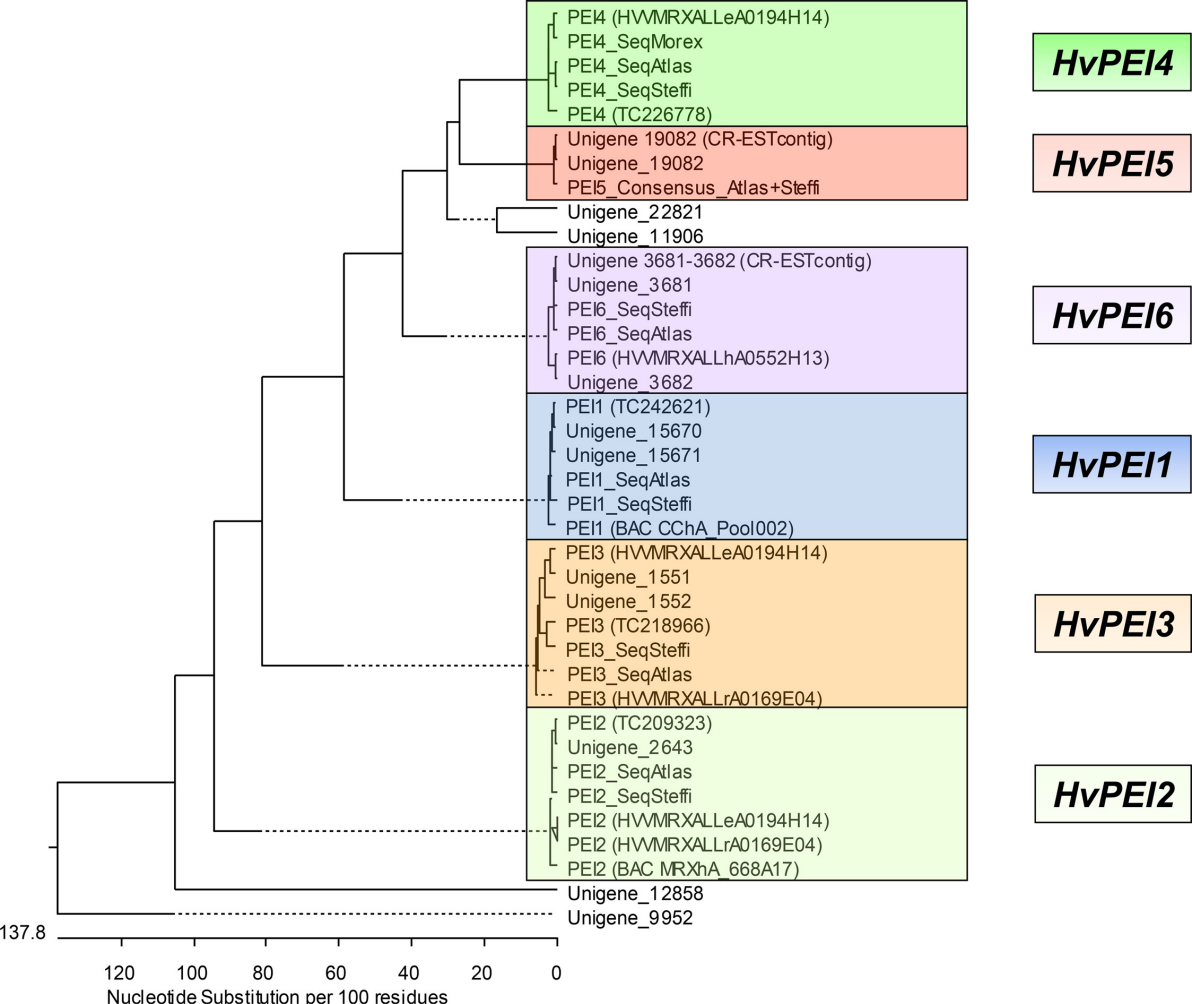
Cluster analysis of the *PEI* gene family in barley. Phylogenetic analysis of six detected *PEI* family members was performed by using database information of known expressed unigenes (HarvEST Assembly 35), BAC sequence information as well as sequence information from 41 analyzed barley cultivars. In addition data from the Gene Index Project (Barley database 12.0) were also included.

Cluster analysis revealed a defined structure of the family of *PECTIN ESTERASE INHIBITOR* genes found in the *Rrs*2 region. By combining database information from different sources (e.g. http://www.harvest-web.org), we were able to identify corresponding expressed unigenes or cDNA clones for a total of six *PEI* genes (Fig 2).

**Fig 3:**
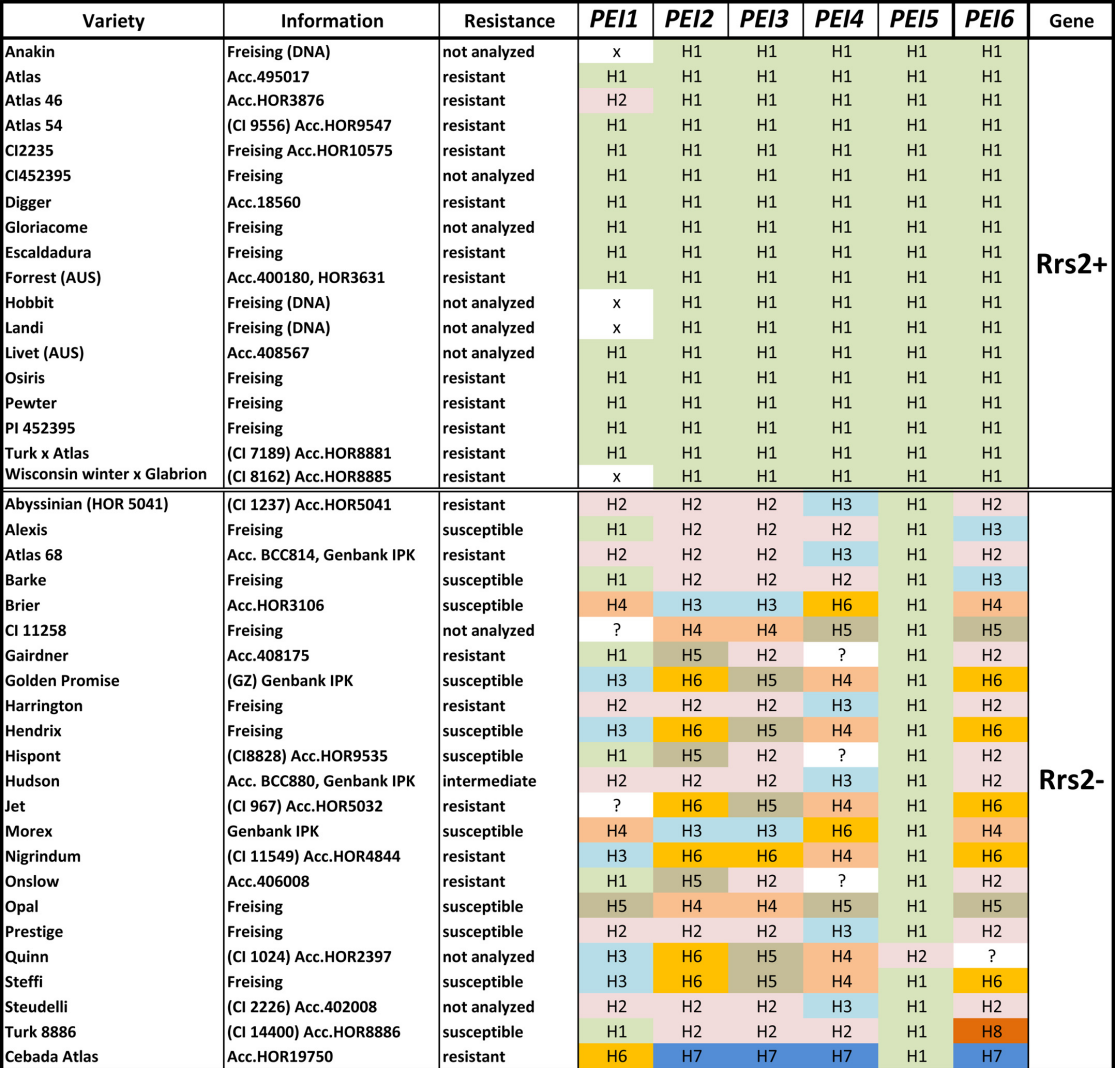
Haplotype analysis of detected *PEI* genes on chromosome 7HS. Full-length sequences of all detected *PECTIN ESTERASE INHIBITOR* members were analyzed in different barley varieties either carrying the *Rrs2* resistance gene or not. Information regarding susceptibility to *Rhynchosporium commune* is according to [29].

**Fig 4:**
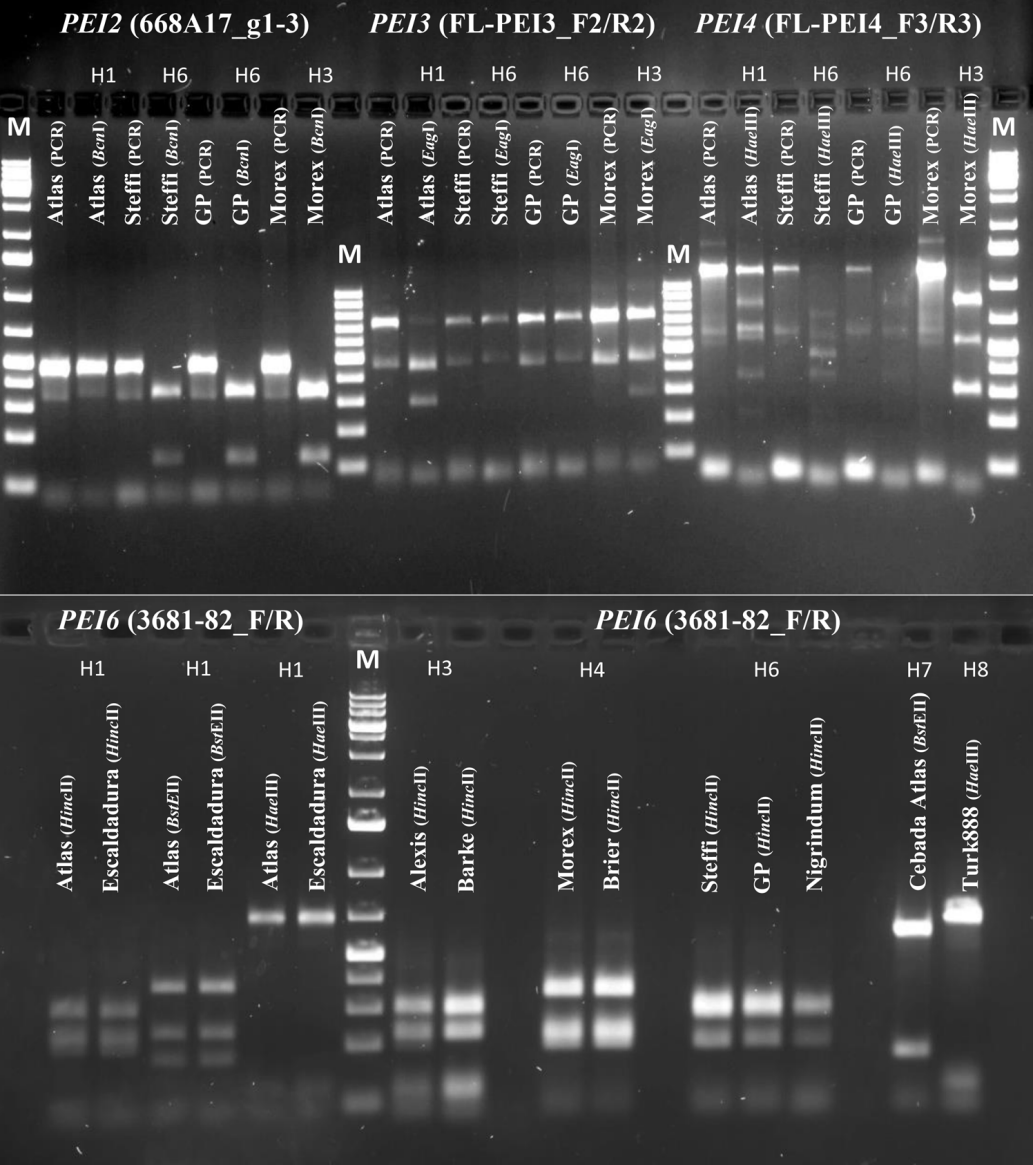
CAPS-markers for different *PEI* genes. As a result of haplotype analysis various SNPs were discovered within the ORF of *HvPEI2*, *HvPEI3*, *HvPEI4* and *HvPEI6*. Polymorphisms were used for CAPS-marker development that in turn could be applied for haplotype discrimination. Examples on the gel photo illustrate results of PCR amplification as well as digestion of the amplicon, with the appropriate restriction enzyme (given in brackets), for different *PEI* family members. As an exception for *HvPEI6* only the results of digestion are presented. Primers that were used for each PCR reaction are given in brackets as well. Additional information regarding distinguished haplotypes is shown as abbreviation on top of lanes with digested fragments (e.g. H1 = haplotype 1, H2 = haplotype 2, et cetera). M = 1kb+ DNA Ladder (Thermo Scientific™ GeneRuler, catalog number #SM1333).

Sequencing of all identified *PEI* genes revealed different haplotypes harboring characteristic SNPs which can be used to distinguish between resistant and susceptible cultivars of barley (Fig 3, S1 Table, S2 File) for the genes *HvPEI2*, *HvPEI3*, *HvPEI4* and *HvPEI6*. One *PEI* candidate gene, *HvPEI4*, also showed insertions as well as deletions in the open reading frame (ORF) in addition (S1 Fig). This could cause a frame shift using the example of cv. ‘Morex’ or an introduction of a premature stop codon in the case of cv. ‘Steffi’. From the obtained sequence information, haplotype-specific CAPS-markers were developed for the genes *HvPEI2*, *HvPEI3*, *HvPEI4*, *HvPEI6* and two additional INDEL-markers for *HvPEI4* (Table 1, Fig 4).

**Table 1:** Haplotype-specific CAPS and INDEL markers for *HvPEI* genes.

Gene	Marker	Primer name	Primer sequence	Enzyme	Atlas (resistant)	Steffi (susceptible)	Haplotypes (resistant and susceptible)	Haplotypes (susceptible)
*HvPEI2*	CAPS	668A17_g1-3F	ACGAACTCAAGGTGGTGGAC	*Bcn*I	660bp	235bp, 425bp	H1	H2, H3, H4 H5, H6, H7
		668A17_g1-3R	TGTTGAGCTCCTGGCTTTCT					
*HvPEI3*	CAPS	FL-PEI3_F2	GCAAGCCTAGCAGCAATGAAAACG	*Eag*I	389bp, 380bp	800bp	H1, H7	H2, H3, H4 H5, H6
		FL-PEI3_R2	TTACATTATATCTTTATTTGCTTTCATACCCCA					
*HvPEI4*	INDEL	P4-INDEL_F2	TGGCATATGATTGTGTTGACGA	-	124bp	128bp	H1, H2, H6, H7	H3, H4, H5
		P4-INDEL_R	GGTGCTACCAAGGCAGTGTTT					
*HvPEI4*	INDEL	PEI4_INDEL2-AT_F	GCGGGCTACGGTCCAATGAG	-	162bp	166bp	H1	H4, H5
		PEI4_INDEL2-AT_R	GTGCTACCAAGGCAGTGTTTAGTTTAAC					
*HvPEI4*	CAPS	FL-PEI4_F3	GTTGCATTAATGAACATGTTGAAGT	*Hae*III	817bp	359bp, 459bp	H1	H7
		FL-PEI4_R3	CGCGGACGGGCCCAAAAC					
*HvPEI4*	CAPS	PEI4_CAPS2_F	CCGGCTAATTTTCCCTACACATCA	*Ban*I	116bp, 123bp	239bp	H1, H2, H3, H4	H5, H6, H7
		PEI4_CAPS2_R	CGTTCTTGCACGCTTCTTCCAT					
*HvPEI6*	CAPS	3681–82_F	CCTACCCAGCGTTCTATATCCATTT	*Nsi*I	615bp	135bp, 480bp	H1	H6
		3681–82_R	CAATCCAAGTGAAGGTAAACAGA					

As exceptional case, *HvPEI5* showed no differences between resistant and susceptible cultivars and hence was not included in further analyses. Although the ViroBLAST (IPK Barley BLAST Server, http://webblast.ipk-gatersleben.de/barley/) search assigned the gene to chromosome 7H we were not able to locate it within the investigated chromosomal region (Fig 1). The gene *HvPEI1* had a presumed susceptible variety-specific haplotype H2 in the *Rrs2* carrying (resistant) variety ‘Atlas46’ and a presumed resistant variety-specific haplotype H1 in several susceptible varieties and was therefore also excluded from further analysis.

### Analysis of transgenic plants

For over-expression of identified *PEI* candidate genes, the barley cv. ‘Golden Promise’ (GP) was used because of its amenability to genetic transformation and its susceptibility to *Rhynchosporium* infection. The full-length coding sequences of *HvPEI2*, *HvPEI3*, *HvPEI4* of resistant cv. ‘Atlas’ were first cloned for each candidate separately into the binary vector p6U and afterwards stably transformed by using *Agrobacterium*-based transformation technology. For *HvPEI2*, *HvPEI3* and *HvPEI4*, 25, 41 and 17 independent primary transgenic plants were generated, respectively. For all investigated *HvPEI2*, *HvPEI3* and *HvPEI4* over-expression (OE)-plants, a significant increase in expression of the respective genes was confirmed by quantitative RT_qPCR in transgenic progeny in comparison to non-transgenic control plants (cv.’Golden Promise’). In several plants with high copy numbers, such as PEI2_OE BG361-E15 lines, the over-expression levels were reduced, which may be an effect of gene silencing. (S2 Fig). The gene *HVPEI6* was only discovered later during the analysis of the respective genomic region; therefore for this gene copy only sequence and haplotype analysis was conducted, but it had not been included in the series of production of transgenic lines which had been finished for the other genes at the time point of discovery of *HvPEI6*.

The copy number in the progeny was assessed by applying TaqMan^™^ PCR using a probe specific for the *HPT* gene. In a T0-plant each transformed gene is usually only present as a single copy per integration site, this means it is present in heterozygous state. Therefore the T1-generation of transformed plants is segregating with respect to copy number, because the transformed sites are not genetically fixed yet. The presence of the transformed *PEI* genes from ‘Atlas’ was furthermore verified by applying gene and allele-specific CAPS-marker analysis.

Several T1 OE-plants for *HvPEI2*, *HvPEI3* and *HvPEI4* were tested for resistance to *R*. *commune* isolate ‘LfL 12F’ in an infection test in the greenhouse at the ‘Bayerische Landesanstalt’ in Freising, Germany. Overall, none of the transformed lines reached the resistance level of the resistant cultivar ‘Atlas’ of 0.78 (Table 2). In a disease scoring range of 0 (most resistant) to 4 (most susceptible) the transformed OE-plants ranged from 2.79 to 3.86 on average. Usually a scoring from 0 to 2 is considered as resistant, while a scoring of 3 and 4 is considered as susceptible according to Jackson and Webster [31]. Comparing the transformed OE-lines to non-transformed ‘Golden Promise’, with an average resistance level of 3.84, several of the transformed OE-lines were significantly different from ‘Golden Promise’ (Table 2). Thereby, four out of five OE-lines for *HvPEI4* reached a significance level of P<0.001. However, none of the transformed plants reached a scoring level to be considered as resistant.

From these results, it can be concluded that none of the *HvPEI2*, *HvPEI3* and *HvPEI4* genes alone seems to represent the *Rrs2* gene of ‘Atlas’, since the resistance level of the OE-lines is rather still in the susceptible range. Some improvements of resistance could be observed, especially with construct *HvPEI4*, however this may be attributed to somaclonal variation. Since the investigated *PEI* genes originate from the susceptible cultivar ‘Morex’, which does not carry *Rrs2*, it is possible that in the resistant cultivar ‘Atlas’ additional genes are present, which were not detected and therefore not tested here. These either could be additional PEI genes or other genes which are not present in ‘Morex’.

**Table 2:** Results of resistance tests with the *Rhynchosporium* isolate LfL12F comparing control plants of non-transformed cv. ‘Golden Promise’ and T_1-_plants of *HvPEI2*, *HvPEI3* and *HvPEI4* over-expression (OE) lines. Since the individual plants of the T_1_-events are still segregating the estimation of copy numbers of the respective gene copies from ‘Atlas’ are indicated: mc = multi-copy, 1c = one copy. The disease scoring ranged from 0 (most resistant) to 4 (most susceptible).

Barley line	Transgene (no. copies)	Mean of disease assessment	Standard deviation	No. of plants	t-test
Golden Promise	-	3.84	0.17	8	
Steffi	-	4	0	7	
Atlas	-	0.78	0.15	8	
BG361_E01	HvPEI2_OE (mc)	3.86	0.26	7	P = 0.914
BG361_E10	HvPEI2_OE (1c)	3.40	0.47	8	P = 0.036*
BG361_E20	HvPEI2_OE (mc)	3.8	0.19	5	P = 0.701
BG432_E05	HvPEI3_OE (1c, mc)	3.25	0.31	8	P<0.001*
BG432/2_E07	HvPEI3_OE (1c)	3.67	0.30	6	P = 0.128
BG432/2_E13	HvPEI3_OE (mc)	3.44	0.39	8	P = 0.025*
BG433/1_E04	HvPEI4_OE (1c, mc)	2.90	0.36	5	P<0.001*
BG433/2_E02	HvPEI4_OE (1c, mc)	3.39	0.13	7	P<0.001*
BG433/1_E02	HvPEI4_OE (mc)	2.79	0.34	7	P<0.001*
BG433/1_E03	HvPEI4_OE (mc)	3.58	0.26	6	P = 0.048*
BG433/2_E03	HvPEI4_OE (mc)	3.10	0.24	4	P<0.001*

*significant (P<0.05) differences between non-transformed ‘Golden Promise’ and transformed OE-lines.

## Discussion

### Involvement of pectin esterase inhibitors in plant defense reactions

Several reports indicate a potential role of *PEI* or *PMEI* genes in plant defense reactions against pathogens:

The *PECTIN METHYL ESTERASE INHIBITOR* gene *CaPMEI1* from pepper (*Capsicum annuum* L.) was isolated and functionally characterized [32,33]. Virus-induced gene silencing of *CaPMEI1* in pepper conferred enhanced susceptibility to *Xanthomonas campestris* pv. *vesicatoria*. Ectopic expression of *CaPMEI1* in Arabidopsis led to enhanced resistance to *Pseudomonas syringae* pv. *tomato*, but not to the biotrophic pathogen *Hyaloperonospora parasitica* [32]. Furthermore, the promoter of *CaPMEI1* induced a high level of GUS reporter activity in tobacco leaf tissue, driven by pathogen infection as well as by ethylene and methyl jasmonate treatment, suggesting an involvement in host defense response [33].

The over-expression of *PMEIs* in Arabidopsis resulted in an increase of 16% of pectin methylesterification in cell walls of the transformed plants [34]. Transformed plants were more resistant to the necrotrophic fungus *Botrytis cinerea*. The reduced symptoms caused by the fungus on transgenic plants were related to its impaired ability to grow on methylesterified pectins [34].

Differences in the methyl ester distribution of homogalacturons were reported for near-isogenic wheat lines resistant and susceptible to the wheat stem rust fungus *Puccinia graminis* f. sp. *Tritici*, which may result in differences for the enzymatic generation of endogenous suppressors resulting in different outcomes of the host-pathogen interaction [35].

The fruit-specific *Fragaria* × *ananassa PECTIN METHYL ESTERASE FaPME1* was expressed in wild strawberry *Fragaria vesca*, resulting in a reduced degree of esterification of the oligogalacturonides from the transgenic fruits as compared to the wild type fruits [36]. The transgenic *F*. *vesca* lines had constitutively activated pathogen defense response, resulting in higher resistance to the necrotrophic fungus *Botrytis cinerea*. It was speculated if oligogalacturonides released from the cell wall may play a role in eliciting defense responses [36].

Transgenic durum wheat lines expressing the *PMEI* from *Actinidia chinensis* (*AcPMEI*) showed a significant increase in the degree of methyl esterification and a significant reduction of disease symptoms caused by the fungal pathogens *Bipolaris sorokiniana* and *Fusarium graminearum* [37]. The expression of the *TaPMEI* gene in wheat was monitored by RT-PCR and high transcription was detected in salicylic acid and hydrogen peroxidase treatments [38]. An enzyme assay indicated that *PME* was completely inhibited by *TaPMEI*.

From the previous examples, two possibilities for the involvement of PMEIs in plant defense mechanism become obvious. The first is an increase of PMEI resulting in repressed PME activity, reduced de-methylesterification and therefore in highly esterified cell walls, which are less prone to the attack by pectin-degrading enzymes of potential pathogens [32,34,37]. Another possibility is the involvement of elicitor molecules which may be released by partial degradation of cell walls [36] and subsequent elicitation or enhancement of the plant defense response. In the given examples, the plant *PMEIs* seem to interact with the own host *PMEs*. To our knowledge, so far no example exists where a plant *PMEI* interacts with a fungal or microbial *PME*.

The concept of ‘effector-triggered defence’ (ETD) has been suggested for apoplastic fungal pathogens, such as *R*. *commune* [39]. ETD responses are characterized by a relatively slow response to the pathogen, not associated to a fast hypersensitive host cell death response, whereby effectors of apoplastic pathogens are recognized at the cell surface [38].

### Conclusions from own experiments

In previous research, the *Rrs2* gene was placed in a linkage block of suppressed recombination which was found in all investigated varieties carrying *Rrs2* and indicated a common origin for this resistance gene [29]. This linkage block had previously been defined by SNPs of non-specified genomic sequences [29]. The present results of haplotype analysis confirmed that genes *HvPEI2*, *HvPEI3*, *HvPEI4* and *HvPEI6* are present within the linkage block, which made these genes possible candidates for *Rrs2* (Table 1) and the testing of this hypothesis in over-expression plants was initiated. As result, none of the tested candidate genes *HvPEI2*, *HvPEI3* and *HvPEI4* alone conferred a high resistance level in transgenic over-expression plants, though an improvement of the resistance level was observed especially with OE-lines for gene *HvPEI4* (Table 2). Therefore, we consider the following hypotheses about the identity of the *Rrs2* gene:

1. *Rrs2* is identical to *HvPEI6* which has not been tested yet in a transgenic approach.
2. The resistance of *Rrs2* requires the action of two or more *PEI* genes.
3. Since all available sequence information comes from the susceptible cultivar ‘Morex’, it is possible that in resistant cultivars, such as ‘Atlas’ additional *PEI* genes or other genes are present in the *Rrs2* region which cannot be found in ‘Morex’.

In conclusion, the question posed in the title cannot conclusively be answered by the current results. We can conclude, that neither *HvPEI2*, *HvPEI3* or *HvPEI4* alone represents the resistance gene *Rrs2*. However the presence of additional PEI genes in the resistant cultivar ‘Atlas’ cannot be excluded. It is also possible that several linked genes are required for resistance expression.

## Materials and Methods

### Plant material and fungal isolates

For haplotype analysis of detected *PECTIN ESTERASE INHIBITOR* genes within the genomic *Rrs*2 region of barley chromosome 7HS, 41 different accessions of cultivated barley were chosen based on reported *Rrs*2 resistance phenotypes. The majority of varieties were taken from Hanemann et al. [29], whereas the following accessions, ‘Anakin’, ‘Atlas46’ (HOR3876), ‘Hobbit’, ‘Landi’, ‘Gloriacome’, ‘CI452395’ (*Rrs2*+) as well as ‘Barke’, ‘CI11258’, ‘Golden Promise’ (*Rrs2*-) were taken in addition.

Several fungal isolates were employed for practical reasons. These included Sachs 147–1 and LfL12F (both obtained from Günther Schweizer, LfL Freising, Germany) and UK7 (obtained from Wolfgang Knogge, IPB Halle, Germany). In all experiments, the two barley varieties ‘Atlas’ (carrying *Rrs2*) and ‘Steffi’ (susceptible), which were the parents of the original mapping population [29], were included as controls.

### Haplotype analysis and marker development

New putative *PEI* genes were identified using two different strategies: First, a keyword search was performed within the HarvEST barley database (http://www.harvest-web.org), version 1.83 (Assembly 35) and sequences of resulting candidates were verified for chromosome position with the ViroBLAST tool (http://webblast.ipk-gatersleben.de/barley/viroblast.php), applying default BLAST parameter settings [40]. The second approach was based on a software application, an automated annotation system for rice: RiceGAAS ([41]; http://ricegaas.dna.affrc.go.jp/), which possesses programs for gene prediction as well as BLAST search options for identification of homologous sequences and gene ontology predictions. Available sequence information of different BAC contigs was analyzed regarding gene content using the graphical user interface.

All *PEI* candidate genes were amplified by PCR from genomic DNA of cultivar ‘Atlas’ (*Rrs2*+, resistant) using specific forward and reverse primers (S2 Table). Primer development was done with the program PrimerSelect™ which is part of the software suit DNASTAR® Lasergene version 10 (DNASTAR, Inc., Madison, Wisconsin, USA).

Haplotype analysis and alignment of nucleotide sequences was performed with the program Sequencher™ version 5.2.3 (Gene Codes Corporation, Ann Arbor, MI, USA). Alignments were analyzed for presence of restriction sites at SNP positions with the Sol Genomics Network tool (http://solgenomics.net/tools/caps_designer/caps_input.pl) [42].

Restriction digestions of PCR products were carried out in a 10-μl volume using 5 μl of PCR product, 1U of the respective restriction endonuclease and an incubation time of 2h at recommended optimal temperature. The restricted fragments were separated on 1–2% 1 x TAE agarose gels.

### Preparation of over-expression constructs

Sequences of candidate *PEI* genes were amplified by PCR from genomic DNA of cultivar ‘Atlas’ (*Rrs2*+, resistant) using specific forward and reverse primers (S2 Table) and cloned into TOPO vector pCR2.1 (Invitrogen, Karlsruhe, Germany) according to manufacturer’s instruction manual for sequence verification. Afterwards correct sequences were transferred into the entry vector pUBI-ABM (DNA-Cloning-Service, Hamburg, Germany) via a digestion and ligation-based cloning strategy that ensured a proper in-frame orientation of *PEI* candidates. Clones that were tested positive for presence of the gene of interest were used for further cloning. A fragment that includes the maize *UBIQUITIN1* promoter, the gene of interest as well as a transcription termination sequence (*NOPALINE SYNTHASE* terminator) was transferred into destination vector p6U (DNA-Cloning-Service, Hamburg, Germany) using the rare cutter *Sfi*I, which has a 13 nucleotide recognition site including five variable nucleotides in central position [43]. Barley ‘Golden Promise’ immature embryos were stably transformed with *PEI* over-expression vectors using *Agrobacterium tumefaciens* according to [44,45].

### Transcript abundance and fungal growth (RT-qPCR)

The assessment of candidate gene expression as well as fungal growth, on stably transgenic barley plants (OE), was performed by quantification via reverse transcription real-time PCR as described by Himmelbach et al. [46]. For this purpose, total RNA and DNA were extracted from control or treated primary leaves as well as first and second leaves at different time points after inoculation with *R*. *commune* spores. RNA isolation was performed using TRIzol Reagent (Life Technologies GmbH, Darmstadt, Germany), which is a monophasic solution of phenol, guanidine isothiocyanate and other components and which allows to perform sequential precipitation of RNA, DNA and proteins from a single sample [47]. After homogenization of the samples with TRIzol Reagent, chloroform was added and the homogenate was allowed to separate into a clear upper aqueous layer containing RNA, an interphase, and a red lower organic layer containing DNA and proteins. RNA was precipitated from the aqueous layer using isopropanol. The clear stratification in diverse phases prevented the contamination with genomic DNA. In case of analysis of target gene expression, cDNA was synthesized from 1 μg total RNA by using the High-Capacity cDNA Reverse Transcription Kit (Applied Biosystems, Foster City, CA, USA) in a 20 μl reaction.

An aliquot of 1 μl cDNA or DNA was taken as template for real-time PCR on a 7900HT Fast Real-Time PCR System (Applied Biosystems, Foster City, CA, USA) by using the GoTaq® qPCR Master Mix Kit (Promega) according to the manufacturer’s manual, except that the total reaction volume was reduced to 10 μl. The primer pairs specific for the respective genes of interest (qRT2-PEI2_F/R, qRT-PEI3_F7/R7, qRT-PEI4_Fa/R) were designed by the software program Lasergene (DNASTAR) (S3 Table) and deployed in a final concentration of 1 μM. For amplification of DNA, a standard two-step, 40-cycle program, followed by a dissociation step was used. After initial incubation at 50°C (2 min) and extended activation of the polymerase at 95°C (10 min), 40 cycles with 95°C (15 sec) and 62°C (30 sec) were applied.

Transcript as well as DNA abundance was determined relative to standard curves and normalized to a gene encoding the barley *UBIQUITIN-CONJUGATING ENZYME* (*UBC*). Data analysis was performed with the sequence detection software (Version 2.2.2; Applied Biosystems) that is provided with the Fast Real-time cycler. Statistical validation was ensured by three biological replicates.

The verification of transgenic barley plants was carried out both via TaqMan® PCR for copy number estimation and RT-qPCR for candidate gene expression. The latter method was already described above whereas the multiplex real-time PCR (TaqMan®) was performed according to Yang et al. [48] by adapting the SYBR Green assay to TaqMan® chemistry: The amplification reaction (15 μl) contained 1 μl genomic DNA, 7.5 μl Maxima Probe/ROX qPCR Master Mix (Thermo Scientific), and forward and reverse primers that are specific for the *HYGROMYCIN PHOSPHOTRANSFERASE* selectable marker gene (*HPT*) or the barley *UBC* gene, respectively. Probes were dual-labeled with a fluorescent reporter dye attached to the 5’-end of the oligonucleotide and the highly efficient dark quencher BHQ1 (Biosearch Technologies, Inc., Petaluma, California, USA) attached to the 3’-end (S3 Table). In case of *HPT* the 6-carboxy-fluoroscein (FAM™) was used, whereas for *UBC* 6-carboxy-4',5'-dichloro-2',7'-dimethoxyfluoresceine (JOE™) was applied. Final primer concentration was adjusted to 0.5 μM and probe concentration to 0.2 μM.

Amplification conditions were derived from a two-step, 40-cycle program described above that is complemented with a third step at 72°C (30 sec), while omitting the dissociation step. Each sample was quantified in triplicate.

To calculate transgene copy numbers, a quantitative method was used which allowed comparing the absolute quantification reaction for the candidate gene *HPT* with the endogenous reference gene *UBC*. Transgene copy numbers were estimated using a homozygous *HPT*-transgenic control plant, verified as described by [44], as calibrator sample and *UBC* as endogenous control. Each reaction comprised three technical replicates. For statistical analysis the software package SigmaPlot11.0 was used.

## Supporting Information

S1 Fig Haplotypes of *HvPEI3*, *HvPEI4* and *HvPEI6*. (PDF)

S2 Fig Transcript abundances in OE-lines for *HvPEI2*, *HvPEI3* and *HvPEI4* in comparison to control lines. (PDF)

S1 File Gene ontology annotations obtained for genes *HvPEI1 –HvPEI6*. (PDF)

S2 File Sequences of genes *HvPEI1* –*HvPEI6* and respective haplotypes. SNPs are labeled in red.
(PDF)

S1 Table Haplotypes and SNPs for five *PEI* genes sequenced in 18 resistant and 23 susceptible cultivars. (XLS)

S2 Table Primer for amplification of full-length sequences of six *PEI* genes in barley. (PDF)

S3 Table Primer for assessment of transcript abundance transgene copy number determination. (PDF)
