# Supplementary material for: Are PECTIN ESTERASE INHIBITOR Genes Involved in Mediating Resistance to Rhynchosporium commune in Barley?
Source: PLoS One. 2016 Mar 3;11(3):e0150485. doi: 10.1371/journal.pone.0150485 (PMC4777559; doi:10.1371/journal.pone.0150485)
Supplement: S1 Fig — (PDF) [file pone.0150485.s001.pdf]

***PLOS ONE* Supporting Information**

Article title: **Are *PECTIN ESTERASE INHIBITOR* genes involved in mediating resistance to *Rhynchosporium commune* in barley?** Authors: Stephan Marzin, Anja Hanemann, Shailendra Sharma, Götz Hensel, Jochen Kumlehn, Günther Schweizer, Marion S. Röder

The following Supporting Information is available for this article:

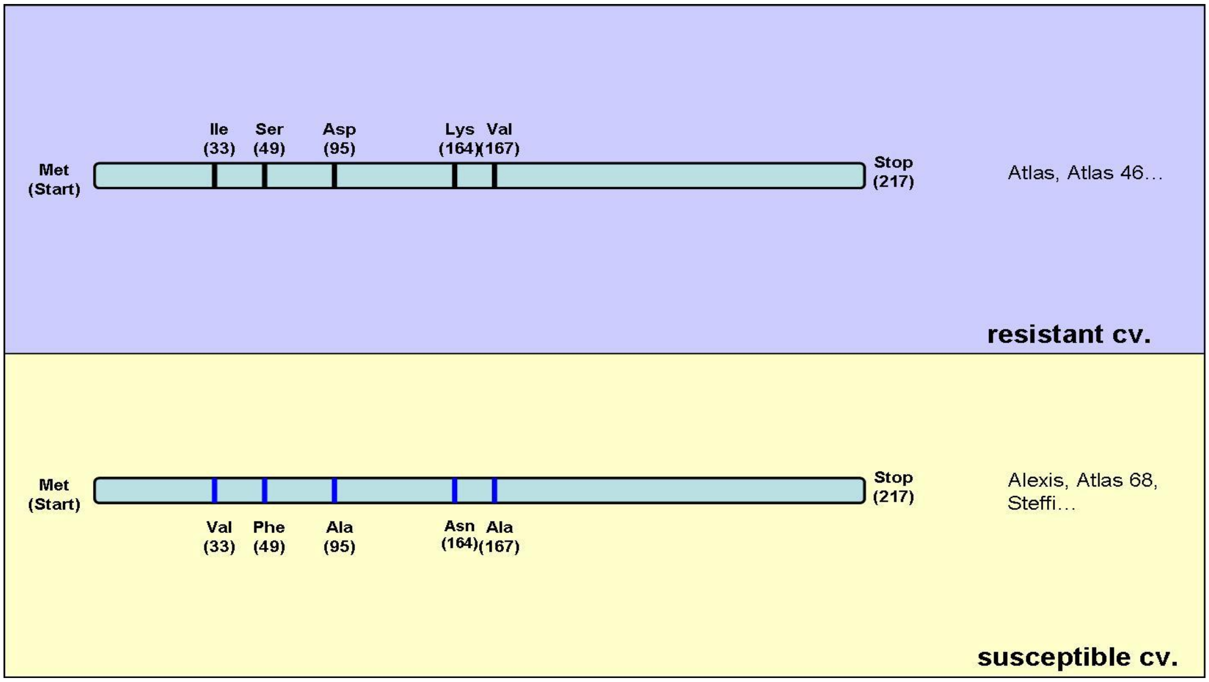

Fig. S1a: Single Nucleotide Polymorphism (SNPs) in the barley gene *HvPEI3*.
Analyses of the full-length sequence of *HvPEI3* reveal a couple of SNPs that lead to amino acid changes and hence differences between resistant and susceptible cultivars.

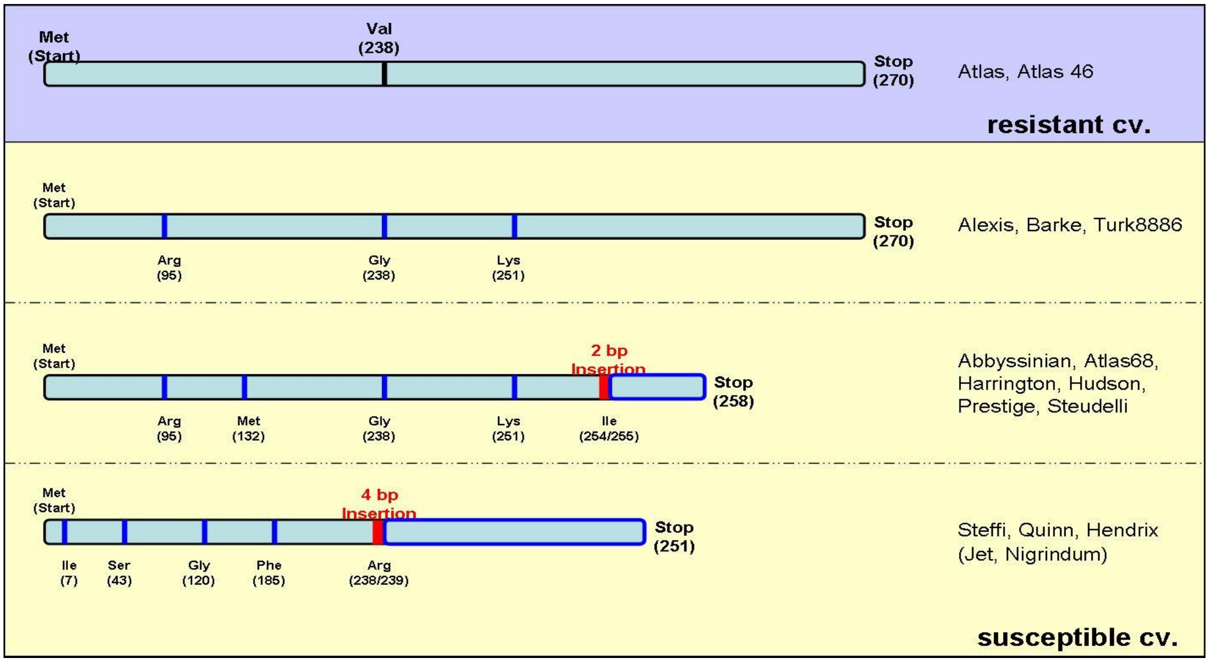

Fig. S1b: Single Nucleotide Polymorphism (SNPs) in the barley gene *HvPEI4*.
Analyses of the full-length sequence of *HvPEI4* reveal a couple of SNPs as well as insertions that lead to amino acid changes or early stop codons and hence differences between resistant and susceptible cultivars.

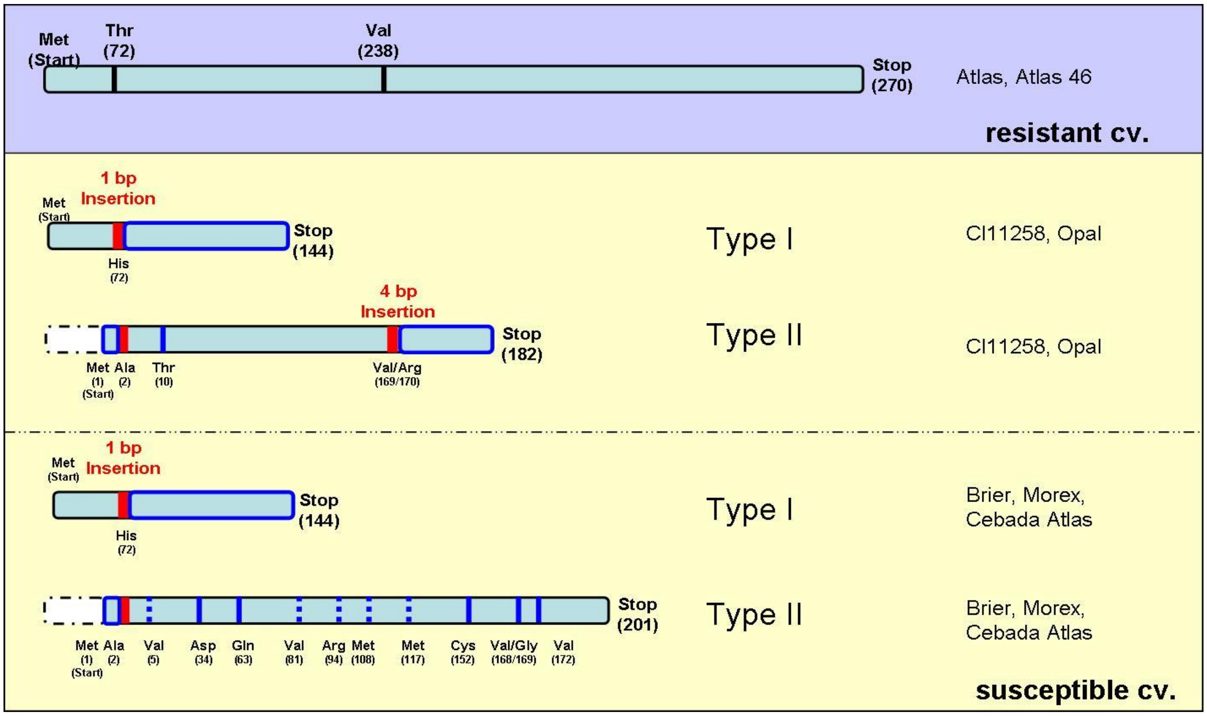

Fig. S1c: Single Nucleotide Polymorphism (SNPs) in the barley gene *HvPEI4*.
Analyses of the full-length sequence of *HvPEI4* reveal a couple of SNPs as well as insertions and/or deletions that lead to amino acid changes or early stop codons and hence differences between resistant and susceptible cultivars.

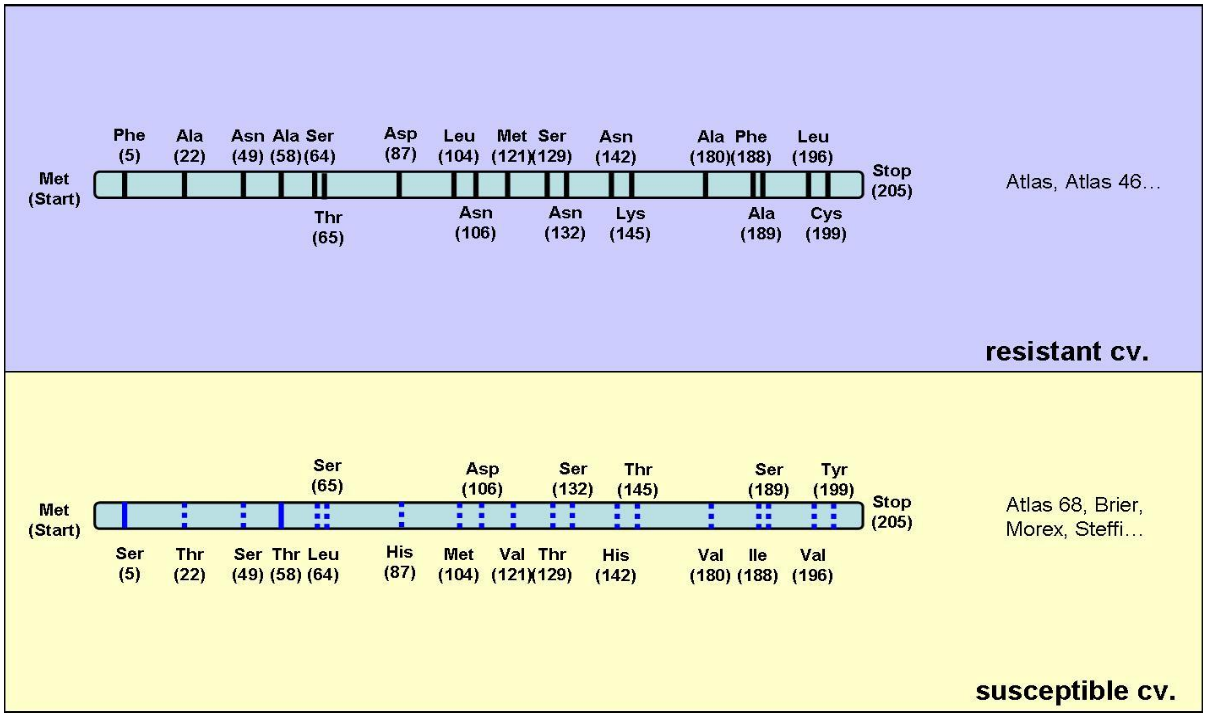

Fig. S1d: Single Nucleotide Polymorphism (SNPs) in the barley gene *HvPEI6*.
Analyses of the full-length sequence of *HvPEI6* reveal a couple of SNPs that lead to amino acid changes and hence differences between resistant and susceptible cultivars.
