# Supplementary material for: Are PECTIN ESTERASE INHIBITOR Genes Involved in Mediating Resistance to Rhynchosporium commune in Barley?
Source: PLoS One. 2016 Mar 3;11(3):e0150485. doi: 10.1371/journal.pone.0150485 (PMC4777559; doi:10.1371/journal.pone.0150485)
Supplement: S2 Fig — (PDF) [file pone.0150485.s002.pdf]

PLOS ONE **Supporting Information**

Article title: **Are *PECTIN ESTERASE INHIBITOR* genes involved in mediating resistance to *Rhynchosporium commune* in barley?**

Authors: Stephan Marzin, Anja Hanemann, Shailendra Sharma, Götz Hensel, Jochen Kumlehn, Günther Schweizer, Marion S. Röder

The following Supporting Information is available for this article:

Supplemental Figure S2

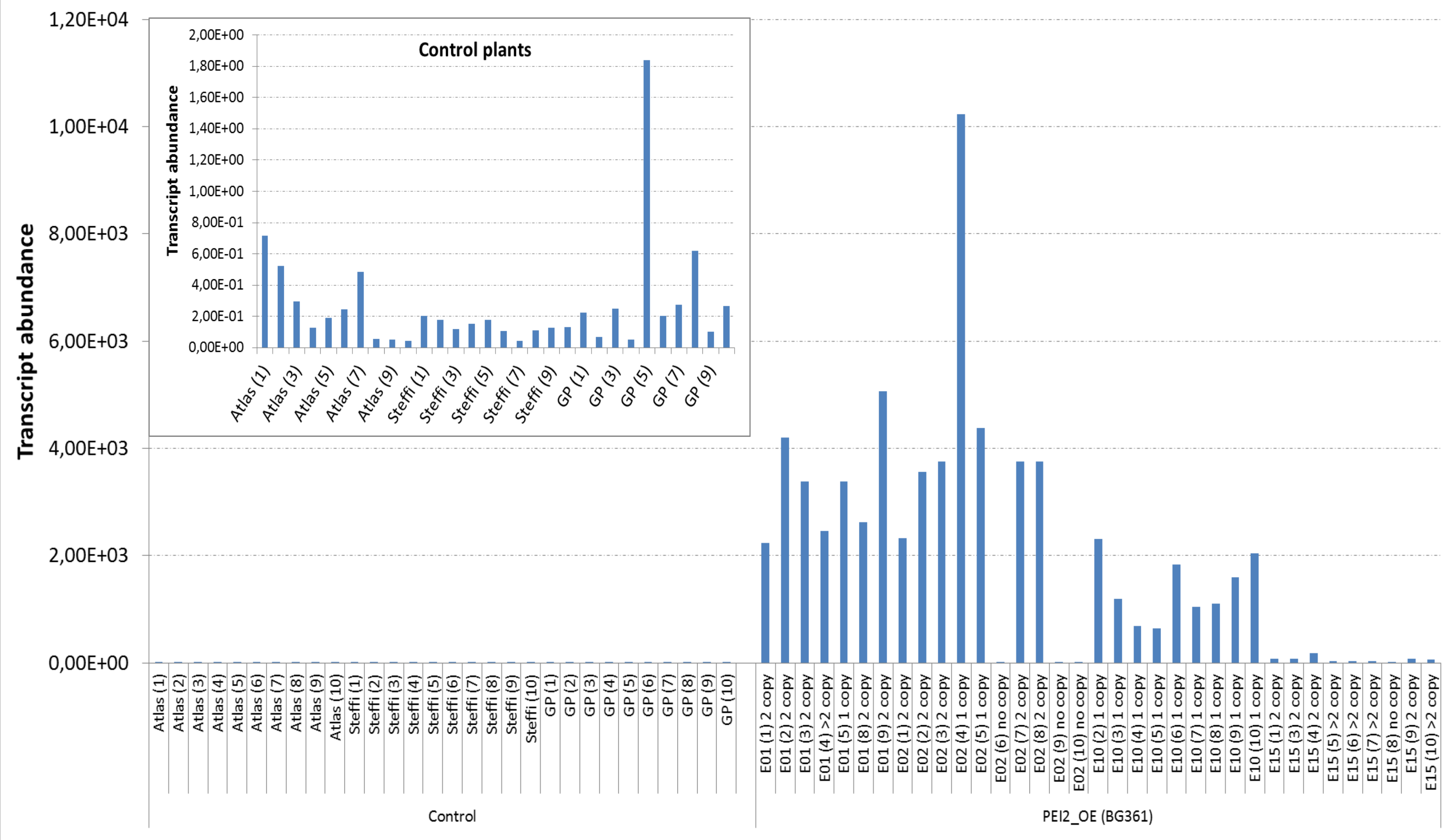

Fig. S2a: Transcript abundance of barley gene *Hv*PEI2.

The figure shows the analysis of gene expression of *Hv*PEI2 between individual non-transformed control plants of resistant cv. Atlas, susceptible cv. Steffi and Golden Promise (details in upper box) as well as transformed Golden Promise OEX-plants. Gene specific primer used for the assessment are shown in Table S3. For assessment of transcript abundance plant material was harvested 14 days after infection with *Rhynchosporium* isolate UK7. Differences were observed between plants carrying 1 or 2 copies (depicted via inscription of y-axis) as well as between plants that carry more than 2 copies [e.g. BG361-E15(5)] or no copies [e.g. BG361-E02(9)], respectively.

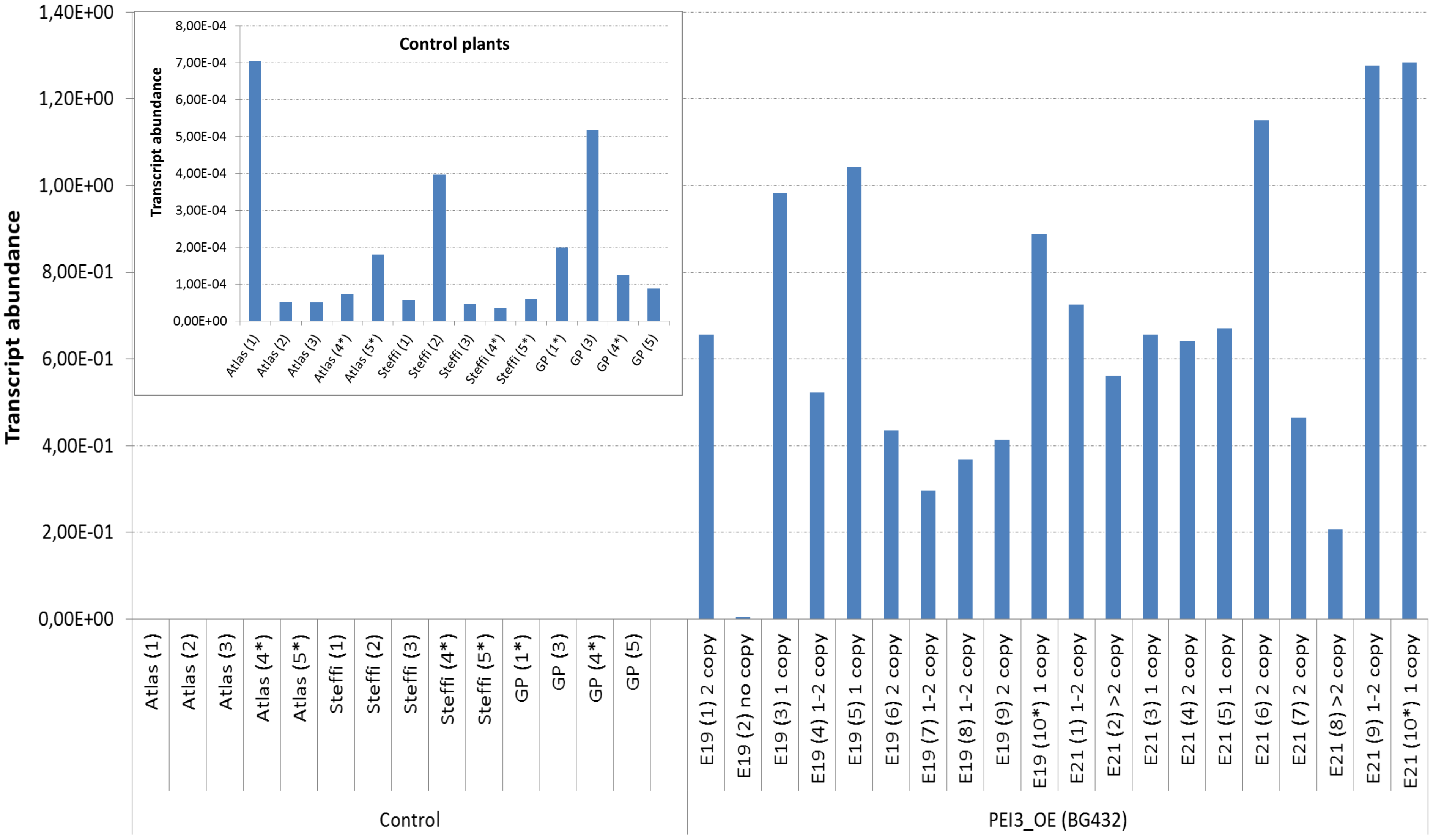

Fig. S2b: Transcript abundance of barley gene *Hv*PEI3.

The figure shows the analysis of gene expression of *Hv*PEI3 between individual non-transformed control plants of resistant cv. Atlas, susceptible cv. Steffi and Golden Promise (details in upper box) as well as transformed Golden Promise OEX-plants. Gene specific primer used for the assessment are shown in Table S3. For assessment of transcript abundance plant material was harvested 14 days after infection with *Rhynchosporium* isolate UK7. Control plants that were not infected with *Rhynchosporium* isolate UK7 are marked with asterisks. Differences were observed between plants carrying 1 or 2 copies (depicted via inscription of y-axis) as well as between plants that carry no copy of the transgene [e.g. BG432-E19(2)].

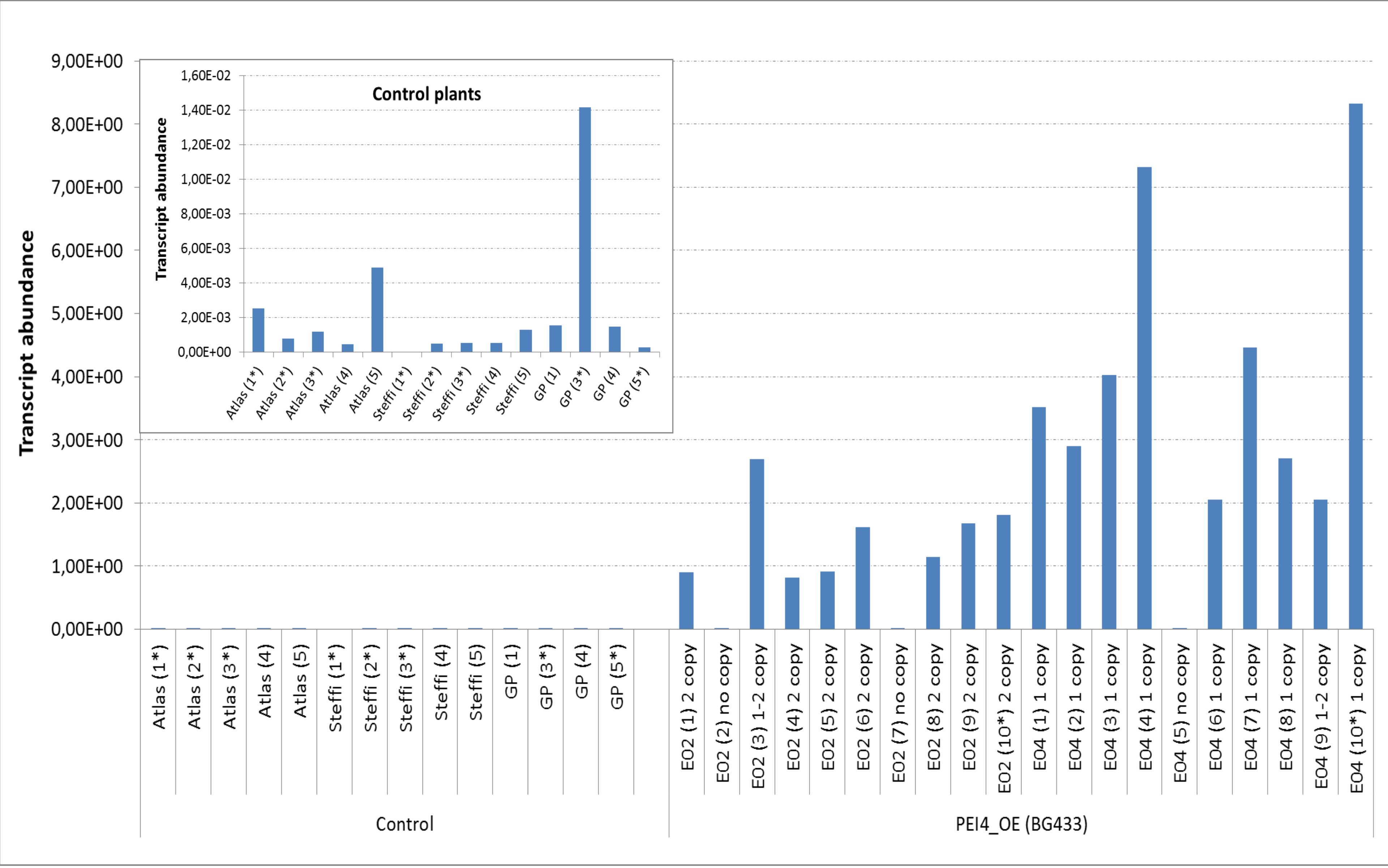

Fig. S2c: Transcript abundance of barley gene *Hv*PEI4.

The figure shows the analysis of gene expression of *Hv*PEI4 between individual non-transformed control plants of resistant cv. Atlas, susceptible cv. Steffi and Golden Promise (details in upper box) as well as transformed Golden Promise OEX-plants. Gene specific primer used for the assessment are shown in Table S3. For assessment of transcript abundance plant material was harvested 14 days after infection with *Rhynchosporium* isolate UK7. Control plants that were not infected with *Rhynchosporium* isolate UK7 are marked with asterisks. Differences were observed between plants carrying 1 or 2 copies (depicted via inscription of y-axis) as well as between plants that carry no copy of the transgene [e.g. BG433-E02(7) or BG433-E04(5)].
