# Supplementary material for: Are PECTIN ESTERASE INHIBITOR Genes Involved in Mediating Resistance to Rhynchosporium commune in Barley?
Source: PLoS One. 2016 Mar 3;11(3):e0150485. doi: 10.1371/journal.pone.0150485 (PMC4777559; doi:10.1371/journal.pone.0150485)
Supplement: S1 File — (PDF) [file pone.0150485.s003.pdf]

PLOS ONE Supporting Information

Article title: **Are *PECTIN ESTERASE INHIBITOR* genes involved in mediating resistance to *Rhynchosporium commune* in barley?**

Authors: Stephan Marzin, Anja Hanemann, Shailendra Sharma, Götz Hensel, Jochen Kumlehn, Günther Schweizer, Marion S. Röder

The following Supporting Information is available for this article:

Notes S1 Gene ontology annotations obtained for genes *HvPEI1* – *HvPEI6.*

Gene No. : PEI1Atlas.AutoPredgene01 : length=211(AA)

Method	Member	Description	Start	End	E-Val	Status	InterPro entry	InterPro description	GO description
HMMTigr	TIGR01614	PME_inhib: pectinesterase inhibitor domain	9	207	1.1e-06	T	IPR006501	Pectinesterase inhibitor	Molecular Function: enzyme inhibitor activity (GO:0004857), Molecular Function: pectinesterase activity (GO:0030599)

Gene No. : PEI2Atlas.AutoPredgene01 : length=207(AA)

Method	Member	Description	Start	End	E-Val	Status	InterPro entry	InterPro description	GO description
HMMTigr	TIGR01614	PME_inhib: pectinesterase inhibitor domain	9	204	0.00025	T	IPR006501	Pectinesterase inhibitor	Molecular Function: enzyme inhibitor activity (GO:0004857), Molecular Function: pectinesterase activity (GO:0030599)

Gene No. : PEI3Atlas.AutoPredgene01 : length=216(AA)

Method	Member	Description	Start	End	E-Val	Status	InterPro entry	InterPro description	GO description
Seg	seg	seg	186	199	NA	?	NULL	NULL	
HMMTigr	TIGR01614	PME_inhib: pectinesterase inhibitor domain	7	201	2.5e-06	T	IPR006501	Pectinesterase inhibitor	Molecular Function: enzyme inhibitor activity (GO:0004857), Molecular Function: pectinesterase activity (GO:0030599)

Gene No. : PEI4Atlas.AutoPredgene01 : length=184(AA)

Method	Member	Description	Start	End	E-Val	Status	InterPro entry	InterPro description	GO description
HMMTigr	TIGR01614	PME_inhib: pectinesterase inhibitor domain	8	181	0.00041	T	IPR006501	Pectinesterase inhibitor	Molecular Function: enzyme inhibitor activity (GO:0004857), Molecular Function: pectinesterase activity (GO:0030599)

Gene No. : PEI5Atlas.AutoPredgene01 : length=206(AA)

Method	Member	Description	Start	End	E-Val	Status	InterPro entry	InterPro description	GO description
Seg	seg	seg	2	22	NA	?	NULL	NULL	

Gene No. : PEI6Atlas.AutoPredgene01 : length=204(AA)

Method	Member	Description	Start	End	E-Val	Status	InterPro entry	InterPro description	GO description
HMMTigr	TIGR01614	PME_inhib: pectinesterase inhibitor domain	4	201	0.00032	T	IPR006501	Pectinesterase inhibitor	Molecular Function: enzyme inhibitor activity (GO:0004857), Molecular Function: pectinesterase activity (GO:0030599)
ScanRegExp	PS00211	ABC_TRANSPORTER_1	188	202	NA	?	IPR003439	ABC transporter related	Molecular Function: ATP binding (GO:0005524), Molecular Function: ATPase activity (GO:0016887)
