# Supplementary material for: Are PECTIN ESTERASE INHIBITOR Genes Involved in Mediating Resistance to Rhynchosporium commune in Barley?
Source: PLoS One. 2016 Mar 3;11(3):e0150485. doi: 10.1371/journal.pone.0150485 (PMC4777559; doi:10.1371/journal.pone.0150485)
Supplement: S2 File — SNPs are labeled in red. (PDF) [file pone.0150485.s004.pdf]

PLOS ONE **Supporting Information**

Article title: **Are *PECTIN ESTERASE INHIBITOR* genes involved in mediating resistance to *Rhynchosporium commune* in barley?**

Authors: Stephan Marzin, Anja Hanemann, Shailendra Sharma, Götz Hensel, Jochen Kumlehn, Günther Schweizer, Marion S. Röder

The following Supporting Information is available for this article:

Notes S2 Sequences of genes *HvPEI1* – *HvPEI6* and respective haplotypes. SNPs are labeled in red.

*Hv*PEI1

CCACTCATACATTCAATAAAATCCAAGGTTTCTAACCGGCCTTGCAACAATGGCAAT
AGCACAGACGACCACCATCTTCTTCTCCGCCATCATCATCGTGCTCTTTTCCTCATC
AATTGTTGCTCATACTAGCGG**(T/C)**GACGTAGGTGGCATGCCAAAAGCAACCGATT
TCATGAC**(A/G)**GCAGCGTGCAAGAACGCTTCAGCCAACT**(T/G)**CGACGAAGA
(T/A)ATCCACATTACCGAAAAATTCTGTGTCTCGACCCTTCGGGCGGACAACCGGA
GCC**(A/G)**CAAGGCTAAGGACCTTCGTGACCTGGCACTCATCGGC**(G/A)**TAGACATC
CTCAAAGCACGTGTCGTCACAGCCAATGGTAAGGTCCAAGAAATGTTGCACGATG
CCAAAAAAGGCAC**(A/G)**TCGATGGCGCGCCACCTCACTGTCTGCAAGCTAGTGTAT
GAAGCAAC**(A/G)(G/A)**TTACC**(T/A)**TCACGAACGTCTGCACTACGTTGCTGAAGGAC
TACCGTGGCCCTAACGG**(C/T)**GGTGACAACGATGGACCGGCATCTGATGACCTCC
CTGAATGTGT**(A/G)**GAGCATGTGAACACCCTCATCG**(A/G)**TTACTGTGGGTATGATA
TTCTTCA**(C/T)(G/A)(T/A)**GTC**(C/G)**GGGCTTCATGCGTTGTTCAACGAAAATCGTGA
GATGAGTAGGCTGGCTTACCTCAACATTGC**(C/G)**TTGCTAGGACCATATAGACACA
AC **(A/G)**ATTCATAATATGTATTT

>Atlas (Haplotype H1)

ATGGCAATAGCACAGACGACCACCATCTTCTTCTCCGCCATCATCATCGTGCTCTT
TTCCTCATCAATTGTTGCTCATACTAGCGG**T**GACGTAGGTGGCATGCCAAAAGCAA
CCGATTTCATGAC**A**GCAGCGTGCAAGAACGCTTCAGCCAACT**T**CGACGAAGA**T**AT
CCACATTACCGAAAAATTCTGTGTCTCGACCCTTCGGGCGGACAACCGGAGCC**A**C
AAGGCTAAGGACCTTCGTGACCTGGCACTCATCGGC**G**TAGACATCCTCAAAGCAC
GTGTCGTCACAGCCAATGGTAAGGTCCAAGAAATGTTGCACGATGCCAAAAAAGG
CAC**A**TCGATGGCGCGCCACCTCACTGTCTGCAAGCTAGTGTATGAAGCAAC**AG**TT
ACC**T**TCACGAACGTCTGCACTACGTTGCTGAAGGACTACCGTGGCCCTAACGG**C**G
GTGACAACGATGGACCGGCATCTGATGACCTCCCTGAATGTGT**A**GAGCATGTGAA
CACCCTCATCG**A**TTACTGTGGGTATGATATTCTTCA**CGT**GTC**C**GGGCTTCATGCGT

TGTTCAACGAAAATCGTGAGATGAGTAGGCTGGCTTACCTCAACATTGCCTTGCTA
GGACCATATAGACACAACAATTCATAATATGTATTT

>Abyssinian (Haplotype H2)

ATGGCAATAGCACAGACGACCACCATCTTCTTCTCCGCCATCATCATCGTGCTCTT
TTCCTCATCAATTGTTGCTCATACTAGCGGTGACGTAGGTGGCATGCCAAAAGCAA
CCGATTTCATGACGGCAGCGTGCAAGAACGCTTCAGCCAACTGCGACGAAGAAAT
CCACATTACCGAAAAATTCTGTGTCTCGACCCTTCGGGCGGACAACCGGAGCCGC
AAGGCTAAGGACCTTCGTGACCTGGCACTCATCGGCATAGACATCCTCAAAGCAC
GTGTCGTCACAGCCAATGGTAAGGTCCAAGAAATGTTGCACGATGCCAAAAAAGG
CACATCGATGGCGCGCCACCTCACTGTCTGCAAGCTAGTGTATGAAGCAACAGTT
ACCTTCACGAACGTCTGCACTACGTTGCTGAAGGACTACCGTGGCCCTAACGGCG
GTGACAACGATGGACCGGCATCTGATGACCTCCCTGAATGTGTAGAGCATGTGAA
CACCCTCATCGATTACTGTGGGTATGATATTCTTCACGTGTCCGGGCTTCATGCGT
TGTTCAACGAAAATCGTGAGATGAGTAGGCTGGCTTACCTCAACATTGCCTTGCTA
GGACCATATAGACACAACAATTCATAATATGTATTT

>Golden Promise (Haplotype H3)

ATGGCAATAGCACAGACGACCACCATCTTCTTCTCCGCCATCATCATCGTGCTCTT
TTCCTCATCAATTGTTGCTCATACTAGCGGTGACGTAGGTGGCATGCCAAAAGCAA
CCGATTTCATGACAGCAGCGTGCAAGAACGCTTCAGCCAACTGCGACGAAGAAAT
CCACATTACCGAAAAATTCTGTGTCTCGACCCTTCGGGCGGACAACCGGAGCCAC
AAGGCTAAGGACCTTCGTGACCTGGCACTCATCGGCGTAGACATCCTCAAAGCAC
GTGTCGTCACAGCCAATGGTAAGGTCCAAGAAATGTTGCACGATGCCAAAAAAGG
CACGTCGATGGCGCGCCACCTCACTGTCTGCAAGCTAGTGTATGAAGCAACGGTT
ACCTTCACGAACGTCTGCACTACGTTGCTGAAGGACTACCGTGGCCCTAACGGCG
GTGACAACGATGGACCGGCATCTGATGACCTCCCTGAATGTGTGGAGCATGTGAA
CACCCTCATCGATTACTGTGGGTATGATATTCTTCATAAGTCGGGGCTTCATGCGT
TGTTCAACGAAAATCGTGAGATGAGTAGGCTGGCTTACCTCAACATTGCCTTGCTA
GGACCATATAGACACAACGATTCATAATATGTATTT

>Morex (Haplotype H4)

ATGGCAATAGCACAGACGACCACCATCTTCTTCTCCGCCATCATCATCGTGCTCTT
TTCCTCATCAATTGTTGCTCATACTAGCGGTGACGTAGGTGGCATGCCAAAAGCAA
CCGATTTCATGACAGCAGCGTGCAAGAACGCTTCAGCCAACTGCGACGAAGAAAT
CCACATTACCGAAAAATTCTGTGTCTCGACCCTTCGGGCGGACAACCGGAGCCAC
AAGGCTAAGGACCTTCGTGACCTGGCACTCATCGGCATAGACATCCTCAAAGCAC
GTGTCGTCACAGCCAATGGTAAGGTCCAAGAAATGTTGCACGATGCCAAAAAAGG
CACATCGATGGCGCGCCACCTCACTGTCTGCAAGCTAGTGTATGAAGCAACAATT
ACCATCACGAACGTCTGCACTACGTTGCTGAAGGACTACCGTGGCCCTAACGGCG
GTGACAACGATGGACCGGCATCTGATGACCTCCCTGAATGTGTAGAGCATGTGAA
CACCCTCATCGATTACTGTGGGTATGATATTCTTCATGTGTCGGGGCTTCATGCGT
TGTTCAACGAAAATCGTGAGATGAGTAGGCTGGCTTACCTCAACATTGCCTTGCTA
GGACCATATAGACACAACAATTCATAATATGTATTT

>Opal (Haplotype H5)

ATGGCAATAGCACAGACGACCACCATCTTCTTCTCCGCCATCATCATCGTGCTCTT
TTCCTCATCAATTGTTGCTCATACTAGCGGCGACGTAGGTGGCATGCCAAAAGCAA
CCGATTTCATGACAGCAGCGTGCAAGAACGCTTCAGCCAACTGCGACGAAGAAAT
CCACATTACCGAAAAATTCTGTGTCTCGACCCTTCGGGCGGACAACCGGAGCCAC
AAGGCTAAGGACCTTCGTGACCTGGCACTCATCGGCGTAGACATCCTCAAAGCAC
GTGTCGTCACAGCCAATGGTAAGGTCCAAGAAATGTTGCACGATGCCAAAAAAGG
CACGTCGATGGCGCGCCACCTCACTGTCTGCAAGCTAGTGTATGAAGCAACGGTT
ACCTTCACGAACGTCTGCACTACGTTGCTGAAGGACTACCGTGGCCCTAACGGCG
GTGACAACGATGGACCGGCATCTGATGACCTCCCTGAATGTGTGGAGCATGTGAA
CACCCTCATCGGTTACTGTGGGTATGATATTCTTCATAAGTCGGGGCTTCATGCGT
TGTTCAACGAAAATCGTGAGATGAGTAGGCTGGCTTACCTCAACATTGCGTTGCTA
GGACCATATAGACACAACAATTCATAATATGTATTT

>CebadaAtlas (Haplotype H6)

ATGGCAATAGCACAGACGACCACCATCTTCTTCTCCGCCATCATCATCGTGCTCTT
TTCCTCATCAATTGTTGCTCATACTAGCGGTGACGTAGGTGGCATGCCAAAAGCAA
CCGATTTCATGACAGCAGCGTGCAAGAACGCTTCAGCCAACTGCGACGAAGAAAT
CCACATTACCGAAAAATTCTGTGTCTCGACCCTTCGGGCGGACAACCGGAGCCAC
AAGGCTAAGGACCTTCGTGACCTGGCACTCATCGGCGTAGACATCCTCAAAGCAC
GTGTCGTCACAGCCAATGGTAAGGTCCAAGAAATGTTGCACGATGCCAAAAAAGG
CACGTCGATGGCGCGCCACCTCACTGTCTGCAAGCTAGTGTATGAAGCAACGGTT
ACCTTCACGAACGTCTGCACTACGTTGCTGAAGGACTACCGTGGCCCTAACGGTG
GTGACAACGATGGACCGGCATCTGATGACCTCCCTGAATGTGTAGAGCATGTGAA
CACCCTCATCGATTACTGTGGGTATGATATTCTTCATGTGTCGGGGCTTCATGCGT
TGTTCAACGAAAATCGTGAGATGAGTAGGCTGGCTTACCTCAACATTGCCTTGCTA
GGACCATATAGACACAACAATTCATAATATGTATTT

*Hv*PEI2

CTTGCAGCAATGGCAACTGC(G/A)CGGGT(A/T)ACCGCTGCCTTCTTCT(C/T)TGTTG
CCGTCATGCTCCTTTTTGTGTCCATCGGCGCTCATGCCGGTGGCCAAGGCGACGA
ACTCAAGGTGGTGGACATCGT(C/G)GTGGAAACCTGCAAGAACGCTTCGAGCAGC
TGCCGCAACAGGCACCTGAACGTCACCCAGGAATTCTGCGTGCAGACTCTCCGGT
C(G/A)GACAAAAGGA(G/T)CTCC(A/G)GGGCCAAGGACCTCCTTGACCTGTCGCTCA
TCGCCGTCGACATCCTCAAGATTCGCGTGGCGGCTGC(C/G)GGTGGCAAGGTGAA
GGAAGCACTCCAGAAAGCCAAGAAAGGTTCGGAGGAGGCGCTCGGCCTCAGGTA
TTGCCAGGTGGACTATGATGTGGCGG(T/C)CCGTACCCTCGGCCTCTGCGACGCC
ATGCTCAGGGACTTCCATGTCCCCAGGGGCGAGGATGAAGACGGCCCGTGGCCT
TTCGAACTGCCCGATTGT(G/A)TGGAGACGG(C/T)GACTGGCCATGTCAGCGACTG
CTGGGG(T/A)(A/C)AGCTTCCCTTGGAAAACTACGCACT(T/.)ATCAAAGAAAGCCAG
GAGCTCAACAAGCTGGGCGACCTGAACAATGCCTTGCTAGGGCCATATGAC(G/T)T
TAATAGT(T/.)A(A/.)(T/G)AATCTATTTTGTGCCAGAACACAGT

>Atlas (Haplotype H1)

ATGGCAACTGCGCGGGTAACCGCTGCCTTCTTCTCTGTTGCCGTCATGCTCCTTTT
TGTGTCCATCGGCGCTCATGCCGGTGGCCAAGGCGACGAACTCAAGGTGGTGGA
CATCGTCGTGGAAACCTGCAAGAACGCTTCGAGCAGCTGCCGCAACAGGCACCTG
AACGTCACCCAGGAATTCTGCGTGCAGACTCTCCGGTCGGACAAAAGGAGCTCCA
GGGCCAAGGACCTCCTTGACCTGTCGCTCATCGCCGTCGACATCCTCAAGATTCG
CGTGGCGGCTGCCGGTGGCAAGGTGAAGGAAGCACTCCAGAAAGCCAAGAAAGG
TTCGGAGGAGGCGCTCGGCCTCAGGTATTGCCAGGTGGACTATGATGTGGCGGT
CCGTACCCTCGGCCTCTGCGACGCCATGCTCAGGGACTTCCATGTCCCCAGGGG
CGAGGATGAAGACGGCCCGTGGCCTTTCGAACTGCCCGATTGTGTGGAGACGGC
GACTGGCCATGTCAGCGACTGCTGGGGTAAGCTTCCCTTGGAAAACTACGCACTT
ATCAAAGAAAGCCAGGAGCTCAACAAGCTGGGCGACCTGAACAATGCCTTGCTAG
GGCCATATGACGTTAATAGTTAATAATCTATTTTG

>Abyssinian (Haplotype H2)

ATGGCAACTGCGCGGGTAACCGCTGCCTTCTTCTCTGTTGCCGTCATGCTCCTTTT
TGTGTCCATCGGCGCTCATGCCGGTGGCCAAGGCGACGAACTCAAGGTGGTGGA
CATCGTCGTGGAAACCTGCAAGAACGCTTCGAGCAGCTGCCGCAACAGGCACCTG
AACGTCACCCAGGAATTCTGCGTGCAGACTCTCCGGTCGGACAAAAGGAGCTCCG
GGGCCAAGGACCTCCTTGACCTGTCGCTCATCGCCGTCGACATCCTCAAGATTCG
CGTGGCGGCTGCCGGTGGCAAGGTGAAGGAAGCACTCCAGAAAGCCAAGAAAGG
TTCGGAGGAGGCGCTCGGCCTCAGGTATTGCCAGGTGGACTATGATGTGGCGGT
CCGTACCCTCGGCCTCTGCGACGCCATGCTCAGGGACTTCCATGTCCCCAGGGG
CGAGGATGAAGACGGCCCGTGGCCTTTCGAACTGCCCGATTGTGTGGAGACGGC
GACTGGCCATGTCAGCGACTGCTGGGGACAGCTTCCCTTGGAAAACTACGCACTT
ATCAAAGAAAGCCAGGAGCTCAACAAGCTGGGCGACCTGAACAATGCCTTGCTAG
GGCCATATGACGTTAATAGTTAATAATCTATTTTG

>Morex (Haplotype H3)

ATGGCAACTGCGCGGGTAACCGCTGCCTTCTTCTCTGTTGCCGTCATGCTCCTTTT
TGTGTCCATCGGCGCTCATGCCGGTGGCCAAGGCGACGAACTCAAGGTGGTGGA
CATCGTGGTGGAAACCTGCAAGAACGCTTCGAGCAGCTGCCGCAACAGGCACCTG
AACGTCACCCAGGAATTCTGCGTGCAGACTCTCCGGTCAGACAAAAGGATCTCCG
GGGCCAAGGACCTCCTTGACCTGTCGCTCATCGCCGTCGACATCCTCAAGATTCG
CGTGGCGGCTGCCGGTGGCAAGGTGAAGGAAGCACTCCAGAAAGCCAAGAAAGG
TTCGGAGGAGGCGCTCGGCCTCAGGTATTGCCAGGTGGACTATGATGTGGCGGT
CCGTACCCTCGGCCTCTGCGACGCCATGCTCAGGGACTTCCATGTCCCCAGGGG
CGAGGATGAAGACGGCCCGTGGCCTTTCGAACTGCCCGATTGTATGGAGACGGC
GACTGGCCATGTCAGCGACTGCTGGGGTAAGCTTCCCTTGGAAAACTACGCACTT
ATCAAAGAAAGCCAGGAGCTCAACAAGCTGGGCGACCTGAACAATGCCTTGCTAG
GGCCATATGACGTTAATAGTTA.GAATCTATTTTG

>CI11258 (Haplotype H4)

ATGGCAACTGC**G**CGGGT**A**ACCGCTGCCTTCTTCT**C**TGTTGCCGTCATGCTCCTTTT
TGTGTCCATCGGCGCTCATGCCGGTGGCCAAGGCGACGAACTCAAGGTGGTGGA
CATCGT**C**GTGGAAACCTGCAAGAACGCTTCGAGCAGCTGCCGCAACAGGCACCTG
AACGTCACCCAGGAATTCTGCGTGCAGACTCTCCGGTC**G**GACAAAAGGA**G**CTCC**G**
GGGCCAAGGACCTCCTTGACCTGTCGCTCATCGCCGTCGACATCCTCAAGATTCG
CGTGGCGGCTGC**G**GGTGGCAAGGTGAAGGAAGCACTCCAGAAAGCCAAGAAAGG
TTCGGAGGAGGCGCTCGGCCTCAGGTATTGCCAGGTGGACTATGATGTGGCGG**C**
CCGTACCCTCGGCCTCTGCGACGCCATGCTCAGGGACTTCCATGTCCCCAGGGG
CGAGGATGAAGACGGCCCGTGGCCTTTCGAACTGCCCGATTGT**G**TGGAGACGG**C**
GACTGGCCATGTCAGCGACTGCTGGGG**TA**AGCTTCCCTTGGAAAACTACGCACT**.**A
TCAAAGAAAGCCAGGAGCTCAACAAGCTGGGCGACCTGAACAATGCCTTGCTAGG
GCCATATGAC**G**TTAATAGT**T**A**A**TAATCTATTTTG

>Gairdner (Haplotype H5)

ATGGCAACTGC**G**CGGGT**A**ACCGCTGCCTTCTTCT**C**TGTTGCCGTCATGCTCCTTTT
TGTGTCCATCGGCGCTCATGCCGGTGGCCAAGGCGACGAACTCAAGGTGGTGGA
CATCGT**C**GTGGAAACCTGCAAGAACGCTTCGAGCAGCTGCCGCAACAGGCACCTG
AACGTCACCCAGGAATTCTGCGTGCAGACTCTCCGGTC**G**GACAAAAGGA**G**CTCC**G**
GGGCCAAGGACCTCCTTGACCTGTCGCTCATCGCCGTCGACATCCTCAAGATTCG
CGTGGCGGCTGC**C**GGTGGCAAGGTGAAGGAAGCACTCCAGAAAGCCAAGAAAGG
TTCGGAGGAGGCGCTCGGCCTCAGGTATTGCCAGGTGGACTATGATGTGGCGG**T**
CCGTACCCTCGGCCTCTGCGACGCCATGCTCAGGGACTTCCATGTCCCCAGGGG
CGAGGATGAAGACGGCCCGTGGCCTTTCGAACTGCCCGATTGT**G**TGGAGACGG**T**
GACTGGCCATGTCAGCGACTGCTGGGG**AC**AGCTTCCCTTGGAAAACTACGCACT**T**
ATCAAAGAAAGCCAGGAGCTCAACAAGCTGGGCGACCTGAACAATGCCTTGCTAG
GGCCATATGAC**G**TTAATAGT**T**A**A**TAATCTATTTTG

>Steffi (Haplotype H6)

ATGGCAACTGC**A**CGGGT**T**ACCGCTGCCTTCTTCT**C**TGTTGCCGTCATGCTCCTTTT
TGTGTCCATCGGCGCTCATGCCGGTGGCCAAGGCGACGAACTCAAGGTGGTGGA
CATCGT**C**GTGGAAACCTGCAAGAACGCTTCGAGCAGCTGCCGCAACAGGCACCTG
AACGTCACCCAGGAATTCTGCGTGCAGACTCTCCGGTC**G**GACAAAAGGA**G**CTCC**G**
GGGCCAAGGACCTCCTTGACCTGTCGCTCATCGCCGTCGACATCCTCAAGATTCG
CGTGGCGGCTGC**C**GGTGGCAAGGTGAAGGAAGCACTCCAGAAAGCCAAGAAAGG
TTCGGAGGAGGCGCTCGGCCTCAGGTATTGCCAGGTGGACTATGATGTGGCGG**T**
CCGTACCCTCGGCCTCTGCGACGCCATGCTCAGGGACTTCCATGTCCCCAGGGG
CGAGGATGAAGACGGCCCGTGGCCTTTCGAACTGCCCGATTGT**G**TGGAGACGG**C**
GACTGGCCATGTCAGCGACTGCTGGGG**TA**AGCTTCCCTTGGAAAACTACGCACT**T**
ATCAAAGAAAGCCAGGAGCTCAACAAGCTGGGCGACCTGAACAATGCCTTGCTAG
GGCCATATGAC**T**TTAATAGT**A**A**A**TAATCTATTTTG

>CebadaAtlas (Haplotype H7)

ATGGCAACTGCACGGGTAACCGCTGCCTTCTTCTTTGTTGCCGTCATGCTCCTTTT
TGTGTCCATCGGCGCTCATGCCGGTGGCCAAGGCGACGAACTCAAGGTGGTGGA
CATCGTCGTGGAAACCTGCAAGAACGCTTCGAGCAGCTGCCGCAACAGGCACCTG
AACGTCACCCAGGAATTCTGCGTGCAGACTCTCCGGTCGGACAAAAGGAGCTCCG
GGGCCAAGGACCTCCTTGACCTGTCGCTCATCGCCGTCGACATCCTCAAGATTCG
CGTGGCGGCTGCCGGTGGCAAGGTGAAGGAAGCACTCCAGAAAGCCAAGAAAGG
TTCGGAGGAGGCGCTCGGCCTCAGGTATTGCCAGGTGGACTATGATGTGGCGGT
CCGTACCCTCGGCCTCTGCGACGCCATGCTCAGGGACTTCCATGTCCCCAGGGG
CGAGGATGAAGACGGCCCGTGGCCTTTCGAACTGCCCGATTGTGTGGAGACGGC
GACTGGCCATGTCAGCGACTGCTGGGGTAAGCTTCCCTTGGAAAACTACGCACTT
ATCAAAGAAAGCCAGGAGCTCAACAAGCTGGGCGACCTGAACAATGCCTTGCTAG
GGCCATATGACGTTAATAGTTAATAATCTATTTTG

*Hv*PEI3

ATAGGTATGTACTCCATCT(G/C)AACCCATCCATCTCTGCACTGCCGTTCCATTTGA
TCCATTTTTTATCTCCCTCAGTTACAATATAGGTCTAGCAAGCCTAGCAG(G/C)A
ATGAAAACGGTGCAACCCATCTCCTTAGCATTCTCTGGAA(T/C)TGTCATCATTCTC
CTTTGCACGACCATCACCGCTCAAGCTGC(C/G)(G/A)ACACCAAGCCC(A/G)TAGCA
TCAGGCATAATATTGGAAGCATGCAAGAATGTCTCAAGCGACT(C/T)CTTCAAGGTG
CGCTTCACGCATGACTTCTGTGTATCGACGCTGCAGTC(A/G)GACAACCGAAGC(A/
G)CC(G/A)ACGCGAAGGACTACCGCATCCTAGCTCTCGTGGCCGTCGAC(A/G)CCA
TGAAGTCCCAAGCCA(C/T)(G/T)GCCG(C/T)GACGG(A/C)CAAGGTTGAGAAATTGAT
CCAGGGTGCTACC(A/G)AGGACAAGA(C/T)TGCGACTCGCG(C/T)CCTTGGGTTCTG
TCGAGTGGACTAC(A/G)GTGGCATGGTGAGCACCCTCGAAATCTGCCATGATATCA
TCCAGAGCTTTGAGATCCGCAAAGGCAATGAAGGT(G/C)(T/C)GACGCCCTTCCTCC
TG(C/A)(C/T)TGGTTGCATTGAGAAAACAACCAAAGC(A/C)ATCAA(G/C)GATTGCG(T
/C)TGACAAAACAGAGTTCGC(G/T)(G/T)CTATTTCAGTAGGGCTAATGAAGGAATAT
GAGGACTTATCTATG(C/T)TTGCCAACCTCAGCAGCTCTTTG(C/T)TACTTCT(C/T)TT
(C/T)ATCACCTCACCTCCTCCTCGTGGACTAGGTTTAGATATTCCTAGT(A/G)ACTAG
AACTGTATGATTATGGTTAAAA

>Atlas (Haplotype H1)

ATAGGTATGTACTCCATCTGAACCCATCCATCTCTGCACTGCCGTTCCATTTGATCC
ATTTTTTATCTCCCTCAGTTACAATATAGGTCTAGCAAGCCTAGCAGGAATGAAAAC
GGTGCAACCCATCTCCTTAGCATTCTCTGGAATTGTCATCATTCTCCTTTGCACGAC
CATCACCGCTCAAGCTGCCGACACCAAGCCCATAGCATCAGGCATAATATTGGAA
GCATGCAAGAATGTCTCAAGCGACTCCTTCAAGGTGCGCTTCACGCATGACTTCTG
TGTATCGACGCTGCAGTCAGACAACCGAAGCACCGACGCGAAGGACTACCGCATC
CTAGCTCTCGTGGCCGTCGACACCATGAAGTCCCAAGCCACGGCCGCGACGGAC
AAGGTTGAGAAATTGATCCAGGGTGCTACCAAGGACAAGACTGCGACTCGCGCCC
TTGGGTTCTGTCGAGTGGACTACAGTGGCATGGTGAGCACCCTCGAAATCTGCCA
TGATATCATCCAGAGCTTTGAGATCCGCAAAGGCAATGAAGGTGTGACGCCCTTCC

TCCTGCCTGGTTGCATTGAGAAAACAACCAAAGCAATCAAGGATTGCGTTGACAAA
ACAGAGTTCGCGGCTATTTCAGTAGGGCTAATGAAGGAATATGAGGACTTATCTAT
GCTTGCCAACCTCAGCAGCTCTTTGCTACTTCTCTTCATCACCTCACCTCCTCCTC
GTGGACTAGGTTTAGATATTCCTAGTAACTAGAACTGTATGATTATGGTTAAAA

> Abyssinian (Haplotype H2)

ATAGGTATGTACTCCATCTGAACCCATCCATCTCTGCACTGCCGTTCCATTTGATCC
ATTTTTTATCTCCCTCAGTTACAATATAGGTCTAGCAAGCCTAGCAGCAATGAAAAC
GGTGCAACCCATCTCCTTAGCATTCTCTGGAATTGTCATCATTCTCCTTTGCACGAC
CATCACCGCTCAAGCTGCCAACACCAAGCCCGTAGCATCAGGCATAATATTGGAA
GCATGCAAGAATGTCTCAAGCGACTTCTTCAAGGTGCGCTTCACGCATGACTTCTG
TGTATCGACGCTGCAGTCGGACAACCGAAGCACCAACGCGAAGGACTACCGCATC
CTAGCTCTCGTGGCCGTCGACGCCATGAAGTCCCAAGCCATTGCCGCGACGGCC
AAGGTTGAGAAATTGATCCAGGGTGCTACCAAGGACAAGACTGCGACTCGCGCCC
TTGGGTTCTGTCGAGTGGACTACAGTGGCATGGTGAGCACCCTCGAAATCTGCCA
TGATATCATCCAGAGCTTTGAGATCCGCAAAGGCAATGAAGGTGTGACGCCCTTCC
TCCTGCCTGGTTGCATTGAGAAAACAACCAAAGCAATCAACGATTGCGCTGACAAA
ACAGAGTTCGCTGCTATTTCAGTAGGGCTAATGAAGGAATATGAGGACTTATCTAT
GCTTGCCAACCTCAGCAGCTCTTTGCTACTTCTCTTCATCACCTCACCTCCTCCTC
GTGGACTAGGTTTAGATATTCCTAGTAACTAGAACTGTATGATTATGGTTAAAA

>Morex (Haplotype H3)

ATAGGTATGTACTCCATCTCAACCCATCCATCTCTGCACTGCCGTTCCATTTGATCC
ATTTTTTATCTCCCTCAGTTACAATATAGGTCTAGCAAGCCTAGCAGCAATGAAAAC
GGTGCAACCCATCTCCTTAGCATTCTCTGGAATTGTCATCATTCTCCTTTGCACGAC
CATCACCGCTCAAGCTGCGGACACCAAGCCCGTAGCATCAGGCATAATATTGGAA
GCATGCAAGAATGTCTCAAGCGACTTCTTCAAGGTGCGCTTCACGCATGACTTCTG
TGTATCGACGCTGCAGTCAGACAACCGAAGCGCCGACGCGAAGGACTACCGCAT
CCTAGCTCTCGTGGCCGTCGACGCCATGAAGTCCCAAGCCACGGCCGTGACGGC
CAAGGTTGAGAAATTGATCCAGGGTGCTACCAAGGACAAGATTGCGACTCGCGCC
CTTGGGTTCTGTCGAGTGGACTACAGTGGCATGGTGAGCACCCTCGAAATCTGCC
ATGATATCATCCAGAGCTTTGAGATCCGCAAAGGCAATGAAGGTCCGACGCCCTTC
CTCCTGATTGGTTGCATTGAGAAAACAACCAAAGCCATCAACGATTGCGCTGACAA
AACAGAGTTCGCGTCTATTTCAGTAGGGCTAATGAAGGAATATGAGGACTTATCTA
TGTTTGCCAACCTCAGCAGCTCTTTGTTACTTCTTTTTATCACCTCACCTCCTCCTC
GTGGACTAGGTTTAGATATTCCTAGTGACTAGAACTGTATGATTATGGTTAAAA

>Opal (Haplotype H4)

ATAGGTATGTACTCCATCTGAACCCATCCATCTCTGCACTGCCGTTCCATTTGATCC
ATTTTTTATCTCCCTCAGTTACAATATAGGTCTAGCAAGCCTAGCAGCAATGAAAAC
GGTGCAACCCATCTCCTTAGCATTCTCTGGAATTGTCATCATTCTCCTTTGCACGAC
CATCACCGCTCAAGCTGCCAACACCAAGCCCGTAGCATCAGGCATAATATTGGAA

GCATGCAAGAATGTCTCAAGCGACTTCTTCAAGGTGCGCTTCACGCATGACTTCTG
TGTATCGACGCTGCAGTCAGACAACCGAAGCACCAACGCGAAGGACTACCGCATC
CTAGCTCTCGTGGCCGTCGACACCATGAAGTCCCAAGCCATTGCCGCGACGGCCA
AGGTTGAGAAATTGATCCAGGGTGCTACCAAGGACAAGACTGCGACTCGCGCCCT
TGGGTTCTGTCGAGTGGACTACGGTGGCATGGTGAGCACCCTCGAAATCTGCCAT
GATATCATCCAGAGCTTTGAGATCCGCAAAGGCAATGAAGGTGTGACGCCCTTCCT
CCTGCCTGGTTGCATTGAGAAAACAACCAAAGCAATCAACGATTGCGCTGACAAAA
CAGAGTTCGCTGCTATTTCAGTAGGGCTAATGAAGGAATATGAGGACTTATCTATG
CTTGCCAACCTCAGCAGCTCTTTGCTACTTCTCTTCATCACCTCACCTCCTCCTCGT
GGACTAGGTTTAGATATTCCTAGTAACTAGAACTGTATGATTATGGTTAAAA

>Steffi (Haplotype H5)

ATAGGTATGTACTCCATCTGAACCCATCCATCTCTGCACTGCCGTTCCATTTGATCC
ATTTTTTATCTCCCTCAGTTACAATATAGGTCTAGCAAGCCTAGCAGCAATGAAAAC
GGTGCAACCCATCTCCTTAGCATTCTCTGGAACTGTCATCATTCTCCTTTGCACGA
CCATCACCGCTCAAGCTGCCAACACCAAGCCCGTAGCATCAGGCATAATATTGGA
AGCATGCAAGAATGTCTCAAGCGACTTCTTCAAGGTGCGCTTCACGCATGACTTCT
GTGTATCGACGCTGCAGTCAGACAACCGAAGCACCAACGCGAAGGACTACCGCAT
CCTAGCTCTCGTGGCCGTCGACACCATGAAGTCCCAAGCCATTGCCGCGACGGCC
AAGGTTGAGAAATTGATCCAGGGTGCTACCAAGGACAAGACTGCGACTCGCGTCC
TTGGGTTCTGTCGAGTGGACTACAGTGGCATGGTGAGCACCCTCGAAATCTGCCA
TGATATCATCCAGAGCTTTGAGATCCGCAAAGGCAATGAAGGTGTGACGCCCTTCC
TCCTGCCTGGTTGCATTGAGAAAACAACCAAAGCAATCAACGATTGCGCTGACAAA
ACAGAGTTCGCTGCTATTTCAGTAGGGCTAATGAAGGAATATGAGGACTTATCTAT
GCTTGCCAACCTCAGCAGCTCTTTGCTACTTCTCTTCATCACCTCACCTCCTCCTC
GTGGACTAGGTTTAGATATTCCTAGTAACTAGAACTGTATGATTATGGTTAAAA

>Nigrindum (Haplotype H6)

ATAGGTATGTACTCCATCTGAACCCATCCATCTCTGCACTGCCGTTCCATTTGATCC
ATTTTTTATCTCCCTCAGTTACAATATAGGTCTAGCAAGCCTAGCAGCAATGAAAAC
GGTGCAACCCATCTCCTTAGCATTCTCTGGAACTGTCATCATTCTCCTTTGCACGA
CCATCACCGCTCAAGCTGCCAACACCAAGCCCGTAGCATCAGGCATAATATTGGA
AGCATGCAAGAATGTCTCAAGCGACTTCTTCAAGGTGCGCTTCACGCATGACTTCT
GTGTATCGACGCTGCAGTCAGACAACCGAAGCACCAACGCGAAGGACTACCGCAT
CCTAGCTCTCGTGGCCGTCGACACCATGAAGTCCCAAGCCATTGCCGCGACGGCC
AAGGTTGAGAAATTGATCCAGGGTGCTACCAAGGACAAGACTGCGACTCGCGTCC
TTGGGTTCTGTCGAGTGGACTACAGTGGCATGGTGAGCACCCTCGAAATCTGCCA
TGATATCATCCAGAGCTTTGAGATCCGCAAAGGCAATGAAGGTGTGACGCCCTTCC
TCCTGCCTGGTTGCATTGAGAAAACAACCAAAGCAATCAACGATTGCGCTGACAAA
ACAGAGTTCGCTGCTATTTCAGTAGGGCTAATGAAGGAATATGAGGACTTATCTAT
GCTTGCCAACCTCAGCAGCTCTTTGCTACTTCTCTTCATCACCTCACCTCCTCCTC
GTGGACTAGGTTTAGATATTCCTAGTAACTAGAACTGTATGATTATGGTTAAAA

>CebadaAtlas (Haplotype H7)

ATAGGTATGTACTCCATCT**G**AACCCATCCATCTCTGCACTGCCGTTCCATTTGATCC
ATTTTTTATCTCCCTCAGTTACAATATAGGTCTAGCAAGCCTAGCAG**G**AATGAAAAC
GGTGCAACCCATCTCCTTAGCATTCTCTGGAA**T**TGTCATCATTCTCCTTTGCACGAC
CATCACCGCTCAAGCTGC**CG**ACACCAAGCCC**A**TAGCATCAGGCATAATATTGGAA
GCATGCAAGAATGTCTCAAGCGACT**C**CTTCAAGGTGCGCTTCACGCATGACTTCTG
TGTATCGACGCTGCAGTC**A**GACAACCGAAGC**A**CC**G**ACGCGAAGGACTACCGCATC
CTAGCTCTCGTGGCCGTCGAC**A**CCATGAAGTCCCAAGCCA**CG**GCCG**C**GACGG**A**C
AAGGTTGAGAAATTGATCCAGGGTGCTACC**G**AGGACAAGA**C**TGCGACTCGCG**C**CC
TTGGGTTCTGTCGAGTGGACTAC**A**GTGGCATGGTGAGCACCCTCGAAATCTGCCA
TGATATCATCCAGAGCTTTGAGATCCGCAAAGGCAATGAAGGT**GT**GACGCCCTTCC
TCCTG**CC**TGGTTGCATTGAGAAAACAACCAAAGC**C**ATCAA**G**GATTGCG**T**TGACAAA
ACAGAGTTCGC**GG**CTATTTCAGTAGGGCTAATGAAGGAATATGAGGACTTATCTAT
G**C**TTGCCAACCTCAGCAGCTCTTTG**C**TACTTCT**C**TT**C**ATCACCTCACCTCCTCCTC
GTGGACTAGGTTTAGATATTCCTAGT**A**ACTAGAACTGTATGATTATGGTTAAAA

*Hv*PEI4 (Primer: FL-PEI4_F3/R3)

GAACTCCCATATTTGTGCACAGATTAGACAATGATCATAGGCATATCCAT**(G/A)**ACC
AAGCTGCACATTGCATTTATGAACCCCACTATATGCATTATAAATAAGTCTCAACCT
CACATACATCATATCCGGCTAATTTTCCCTACACATCATTCTATAT**(T/C)**CATTTATCC
ACACCTCTCTAGGCTGTTGTACAATTCAAGGCTTCTAGCAAGCCTTGCAACAATATA
TCACACCAACACAAATGGCACCATCATCTTTTCCACCATTGCCTTAATGCTACTCTC
CGTGACCATGACGGCTCAAGCCGATGGCTCCGGTGGTGGAATGCCCAAGGCGAA
CGAACTTATGGAAGAAGCGTGCAAGAACGCATCAATCAACAACCCCCATGTGG**(A/**
G)CCCTGTCACAGAGGAATTCTGCTTAAGAACCCTCCTGTCAGACAATCGAAGCAT
GGAGGC**(A/G)**AAGGACCTCCGTGAACTGCTGCTAGTCGCCGTCGACATCCTTAGG
GCACGGGTTGCTGCTACCGGCGGCATGGTCAACAAAATGTTGGAGAACACCAGGA
AAGGCACAGTGCCAATGCGTGTCCTCAGTTTTT**(G/T)**TGAAGTTGATTATGAATCCA
TGTTGAGCATCCTTAAGATATGTGATGCCATGATCAGGGACTATCAAGGTGGCGAG
GGCGGGCTACGGTCCAATGAGCTAGCCAGTTATGTGGATATGGCATATGATTGTG
TTGACGAGTGCGGCTCCGAGCTTGACGT**(./A)(./C)(./G)(./G)**TATGCCCCTGGCGGG
GGCTCTTGTCAACGAAAACAATGAATTGGGCATGCTGGTTAAACTAAACACTGCCT
TGGTAGCACCACGTAGGTTTCTTGAGTAATATAATGTTTGTTTTCATTTGGGAAAAA
ATATGTTGATGAGAAATAAAATGTGCTCTTAATAGTAATAATTATAAATATAATAATA
AG

>Atlas (Haplotype H1)

GAACTCCCATATTTGTGCACAGATTAGACAATGATCATAGGCATATCCAT**G**ACCAA
GCTGCACATTGCATTTATGAACCCCACTATATGCATTATAAATAAGTCTCAACCTCA
CATACATCATATCCGGCTAATTTTCCCTACACATCATTCTATAT**T**CATTTATCCACAC
CTCTCTAGGCTGTTGTACAATTCAAGGCTTCTAGCAAGCCTTGCAACAATATATCAC
ACCAACACAAATGGCACCATCATCTTTTCCACCATTGCCTTAATGCTACTCTCCGTG
ACCATGACGGCTCAAGCCGATGGCTCCGGTGGTGGAATGCCCAAGGCGAACGAA
CTTATGGAAGAAGCGTGCAAGAACGCATCAATCAACAACCCCCATGTGG**A**CCCTG
TCACAGAGGAATTCTGCTTAAGAACCCTCCTGTCAGACAATCGAAGCATGGAGGC
AAAGGACCTCCGTGAACTGCTGCTAGTCGCCGTCGACATCCTTAGGGCACGGGTT
GCTGCTACCGGCGGCATGGTCAACAAAATGTTGGAGAACACCAGGAAAGGCACAG

TGCCAATGCGTGTCCTCAGTTTTT**G**TGAAGTTGATTATGAATCCATGTTGAGCATCC
TTAAGATATGTGATGCCATGATCAGGGACTATCAAGGTGGCGAGGGCGGGCTACG
GTCCAATGAGCTAGCCAGTTATGTGGATATGGCATATGATTGTGTTGACGAGTGCG
GCTCCGAGCTTGACGT**....**TATGCCCCTGGCGGGGGCTCTTGTCAACGAAAACAAT
GAATTGGGCATGCTGGTTAAACTAAACACTGCCTTGGTAGCACCACGTAGGTTTCT
TGAGTAATATAATGTTTGTTTTCATTTGGGAAAAAATATGTTGATGAGAAATAAAATG
TGCTCTTAATAGTAATAATTATAAATATAATAATAAG

>Steffi (Haplotype H4)

GAACTCCCATATTTGTGCACAGATTAGACAATGATCATAGGCATATCCAT**A**ACCAA
GCTGCACATTGCATTTATGAACCCCACTATATGCATTATAAATAAGTCTCAACCTCA
CATACATCATATCCGGCTAATTTTCCCTACACATCATTCTATAT**C**CATTTATCCACAC
CTCTCTAGGCTGTTGTACAATTCAAGGCTTCTAGCAAGCCTTGCAACAATATATCAC
ACCAACACAAATGGCACCATCATCTTTTCCACCATTGCCTTAATGCTACTCTCCGTG
ACCATGACGGCTCAAGCCGATGGCTCCGGTGGTGGAATGCCCAAGGCGAACGAA
CTTATGGAAGAAGCGTGCAAGAACGCATCAATCAACAACCCCCATGTGG**G**CCCTG
TCACAGAGGAATTCTGCTTAAGAACCCTCCTGTCAGACAATCGAAGCATGGAGGC
GAAGGACCTCCGTGAACTGCTGCTAGTCGCCGTCGACATCCTTAGGGCACGGGTT
GCTGCTACCGGCGGCATGGTCAACAAAATGTTGGAGAACACCAGGAAAGGCACAG
TGCCAATGCGTGTCCTCAGTTTTT**T**TGAAGTTGATTATGAATCCATGTTGAGCATCC
TTAAGATATGTGATGCCATGATCAGGGACTATCAAGGTGGCGAGGGCGGGCTACG
GTCCAATGAGCTAGCCAGTTATGTGGATATGGCATATGATTGTGTTGACGAGTGCG
GCTCCGAGCTTGACGT**ACGG**TATGCCCCTGGCGGGGGCTCTTGTCAACGAAAACA
ATGAATTGGGCATGCTGGTTAAACTAAACACTGCCTTGGTAGCACCACGTAGGTTT
CTTGAGTAATATAATGTTTGTTTTCATTTGGGAAAAAATATGTTGATGAGAAATAAAA
TGTGCTCTTAATAGTAATAATTATAAATATAATAATAAG

*Hv*PEI4 (Primer: FL-PEI4_F1/R1)

TAGCAAGCCTTGCAACAATATATCACAC**(C/G)**AACACAAATGGC**(./C)**ACCATC**(A/G)**
TCTTTTCCACCATT**(G/A)**C**(C/G)**TTAATGCTACTCTCCGTGACCATGACGGCTCAAG
CCGATGGCTCC**(G/C)**GTGGTGGAATGCCCAAGGCGAACGA**(A/C)**CT**(T/C)**ATGGAA
GAAGCGTGCAAGAACGCATC**(A/G)**ATCAACAACCCCCATGTGG**(A/G)**CCCTGTCAC
AGAGGA**(A/G)**TTCTGCTTAAGAACCCTC**(C/A)(T/A)**GTCAGACAATCGAAGCATGGA
GGC**(A/G/T)**AAGGACCTCCGTGAACTGCTGCTAGTCG**(C/T)**CGTCGACATCCTTAGG
GCACGGGTTGCTGCTACCGGC**(G/C)**GCATGGTCAACAAAATGTTGGAGAACACCA
GGAAAGGCACA**(G/A)**T**(G/A)**CCAATGCG**(T/G/C)**GTCCTCAGTTTTT**(G/T)**TGA**(A/T)**G
TTGATTATGAATCCATGTTGAGCATCCTTAAGATATGTGATGCCATGATCAGGGACT
ATCAAGG**(T/C)**GGCGAGGGCGGGCTACGGTCCAATGAGCTAGCCAGTT**(A/G)**TGTG
GATATGGCATATGATTGTGTTGACGAGTGCGGCTCCGAGCTTG**(A/T)(C/G)**G**(T/G)(./**
A)(./C)(./G)(./G)TATGCCC**(C/G)**TGGCGGGGGCTCTTGTCAACGAAAACAAT**(G/A)**AAT
TGGGCAT**(./A)(./T)**GCTGGTTAAACT**(A/G)**AACACTGCCTT**(G/A)**GTAGCACCACGTA
GGTTTCT**(T/G)**GAGTAATATAATGTTTGTTT

>Atlas (Haplotype H1)

TAGCAAGCCTTGCAACAATATATCACACCAACACAAATGGC.ACCATCATCTTTTCC
ACCATTGCCTTAATGCTACTCTCCGTGACCATGACGGCTCAAGCCGATGGCTCCG
GTGGTGGAATGCCCAAGGCGAACGAACTTATGGAAGAAGCGTGCAAGAACGCATC
AATCAACAACCCCCATGTGGACCCTGTCACAGAGGAATTCTGCTTAAGAACCCTCC
TGTCAGACAATCGAAGCATGGAGGCAAAGGACCTCCGTGAACTGCTGCTAGTCGC
CGTCGACATCCTTAGGGCACGGGTTGCTGCTACCGGCGGCATGGTCAACAAAATG
TTGGAGAACACCAGGAAAGGCACAGTGCCAATGCGTGTCCTCAGTTTTTGTGAAG
TTGATTATGAATCCATGTTGAGCATCCTTAAGATATGTGATGCCATGATCAGGGACT
ATCAAGGTGGCGAGGGCGGGCTACGGTCCAATGAGCTAGCCAGTTATGTGGATAT
GGCATATGATTGTGTTGACGAGTGCGGCTCCGAGCTTGACGT....TATGCCCCTGG
CGGGGGCTCTTGTCAACGAAAACAATGAATTGGGCAT..GCTGGTTAAACTAAACAC
TGCCTTGGTAGCACCACGTAGGTTTCTTGAGTAATATAATGTTTGTTT

>Alexis (Haplotype H2)

TAGCAAGCCTTGCAACAATATATCACACGAACACAAATGGC.ACCATCATCTTTTCC
ACCATTGCCTTAATGCTACTCTCCGTGACCATGACGGCTCAAGCCGATGGCTCCC
GTGGTGGAATGCCCAAGGCGAACGAACTTATGGAAGAAGCGTGCAAGAACGCATC
AATCAACAACCCCCATGTGGACCCTGTCACAGAGGAATTCTGCTTAAGAACCCTCC
TGTCAGACAATCGAAGCATGGAGGCGAAGGACCTCCGTGAACTGCTGCTAGTCGC
CGTCGACATCCTTAGGGCACGGGTTGCTGCTACCGGCGGCATGGTCAACAAAATG
TTGGAGAACACCAGGAAAGGCACAGTACCAATGCGTGTCCTCAGTTTTTGTGAAGT
TGATTATGAATCCATGTTGAGCATCCTTAAGATATGTGATGCCATGATCAGGGACTA
TCAAGGTGGCGAGGGCGGGCTACGGTCCAATGAGCTAGCCAGTTATGTGGATATG
GCATATGATTGTGTTGACGAGTGCGGCTCCGAGCTTGACGG....TATGCCCCTGGC
GGGGGCTCTTGTCAACGAAAACAATAAATTGGGCAT..GCTGGTTAAACTAAACACT
GCCTTGGTAGCACCACGTAGGTTTCTTGAGTAATATAATGTTTGTTT

>Abyssinian (Haplotype H3)

TAGCAAGCCTTGCAACAATATATCACACGAACACAAATGGC.ACCATCATCTTTTCC
ACCATTGCCTTAATGCTACTCTCCGTGACCATGACGGCTCAAGCCGATGGCTCCC
GTGGTGGAATGCCCAAGGCGAACGAACTTATGGAAGAAGCGTGCAAGAACGCATC
AATCAACAACCCCCATGTGGACCCTGTCACAGAGGAATTCTGCTTAAGAACCCTCA
TGTCAGACAATCGAAGCATGGAGGCGAAGGACCTCCGTGAACTGCTGCTAGTCGC
CGTCGACATCCTTAGGGCACGGGTTGCTGCTACCGGCGGCATGGTCAACAAAATG
TTGGAGAACACCAGGAAAGGCACAGTGCCAATGCGTGTCCTCAGTTTTTGTGAAG
TTGATTATGAATCCATGTTGAGCATCCTTAAGATATGTGATGCCATGATCAGGGACT
ATCAAGGTGGCGAGGGCGGGCTACGGTCCAATGAGCTAGCCAGTTATGTGGATAT
GGCATATGATTGTGTTGACGAGTGCGGCTCCGAGCTTGACGG....TATGCCCCTGG
CGGGGGCTCTTGTCAACGAAAACAATAAATTGGGCATATGCTGGTTAAACTAAACA
CTGCCTTGGTAGCACCACGTAGGTTTCTTGAGTAATATAATGTTTGTTT

>Steffi (Haplotype H4)

TAGCAAGCCTTGCAACAATATATCACACCAACACAAATGGC.ACCATCATCTTTTCC
ACCATTGCCTTAATGCTACTCTCCGTGACCATGACGGCTCAAGCCGATGGCTCCG

GTGGTGGAATGCCCAAGGCGAACGAACTTATGGAAGAAGCGTGCAAGAACGCATC
AATCAACAACCCCCATGTGGGCCCTGTCACAGAGGAATTCTGCTTAAGAACCCTC
CTGTCAGACAATCGAAGCATGGAGGCGAAGGACCTCCGTGAACTGCTGCTAGTCG
CCGTCGACATCCTTAGGGCACGGGTTGCTGCTACCGGCGGCATGGTCAACAAAAT
GTTGGAGAACACCAGGAAAGGCACAGTGCCAATGCGTGTCCTCAGTTTTTTTGAA
GTTGATTATGAATCCATGTTGAGCATCCTTAAGATATGTGATGCCATGATCAGGGA
CTATCAAGGTGGCGAGGGCGGGCTACGGTCCAATGAGCTAGCCAGTTATGTGGAT
ATGGCATATGATTGTGTTGACGAGTGCGGCTCCGAGCTTGACGTACGGTATGCCC
CTGGCGGGGGCTCTTGTCAACGAAAACAATGAATTGGGCAT..GCTGGTTAAACTAA
ACACTGCCTTGGTAGCACCACGTAGGTTTCTTGAGTAATATAATGTTTGTTT

>Opal (Haplotype H5)

TAGCAAGCCTTGCAACAATATATCACACCAACACAAATGGCCACCATCATCTTTTCC
ACCATTACCTTAATGCTACTCTCCGTGACCATGACGGCTCAAGCCGATGGCTCCG
GTGGTGGAATGCCCAAGGCGAACGAACTTATGGAAGAAGCGTGCAAGAACGCATC
AATCAACAACCCCCATGTGGACCCTGTCACAGAGGAGTTCTGCTTAAGAACCCTC
CTGTCAGACAATCGAAGCATGGAGGCGAAGGACCTCCGTGAACTGCTGCTAGTCG
CCGTCGACATCCTTAGGGCACGGGTTGCTGCTACCGGCGGCATGGTCAACAAAAT
GTTGGAGAACACCAGGAAAGGCACAGTGCCAATGCGTGTCCTCAGTTTTTGTGAA
GTTGATTATGAATCCATGTTGAGCATCCTTAAGATATGTGATGCCATGATCAGGGA
CTATCAAGGTGGCGAGGGCGGGCTACGGTCCAATGAGCTAGCCAGTTATGTGGAT
ATGGCATATGATTGTGTTGACGAGTGCGGCTCCGAGCTTGACGTACGGTATGCCC
CTGGCGGGGGCTCTTGTCAACGAAAACAATGAATTGGGCAT..GCTGGTTAAACTAA
ACACTGCCTTGGTAGCACCACGTAGGTTTCTTGAGTAATATAATGTTTGTTT

>Morex (Haplotype H6)

TAGCAAGCCTTGCAACAATATATCACACCAACACAAATGGCCACCATCGTCTTTTC
CACCATTGCGTTAATGCTACTCTCCGTGACCATGACGGCTCAAGCCGATGGCTCC
GGTGGTGGAATGCCCAAGGCGAACGACCTCATGGAAGAAGCGTGCAAGAACGCA
TCGATCAACAACCCCCATGTGGACCCTGTCACAGAGGAATTCTGCTTAAGAACCCT
CCAGTCAGACAATCGAAGCATGGAGGCTAAGGACCTCCGTGAACTGCTGCTAGTC
GTCGTCGACATCCTTAGGGCACGGGTTGCTGCTACCGGCGGCATGGTCAACAAAA
TGTTGGAGAACACCAGGAAAGGCACAATGCCAATGCGGGTCCTCAGTTTTTGTGA
TGTTGATTATGAATCCATGTTGAGCATCCTTAAGATATGTGATGCCATGATCAGGGA
CTATCAAGGCGGCGAGGGCGGGCTACGGTCCAATGAGCTAGCCAGTTGTGTGGA
TATGGCATATGATTGTGTTGACGAGTGCGGCTCCGAGCTTGTGGG....TATGCCCGT
GGCGGGGGCTCTTGTCAACGAAAACAATGAATTGGGCAT..GCTGGTTAAACTGAA
CACTGCCTTAGTAGCACCACGTAGGTTTCTGGAGTAATATAATGTTTGTTT

>Cebada Atlas (Haplotype H7)

TAGCAAGCCTTGCAACAATATATCACACCAACACAAATGGCCACCATCATCTTTTCC
ACCATTGCCTTAATGCTACTCTCCGTGACCATGACGGCTCAAGCCGATGGCTCCG
GTGGTGGAATGCCCAAGGCGAACGACCTCATGGAAGAAGCGTGCAAGAACGCAT
CGATCAACAACCCCCATGTGGACCCTGTCACAGAGGAATTCTGCTTAAGAACCCT

C**CA**GTCAGACAATCGAAGCATGGAGGC**T**AAGGACCTCCGTGAACTGCTGCTAGTC
G**C**CGTCGACATCCTTAGGGCACGGGTTGCTGCTACCGGC**C**GCATGGTCAACAAAA
TGTTGGAGAACACCAGGAAAGGCACA**G**T**G**CCAATGCG**C**GTCCTCAGTTTTT**G**TGA
AGTTGATTATGAATCCATGTTGAGCATCCTTAAGATATGTGATGCCATGATCAGGG
ACTATCAAGG**C**GGCGAGGGCGGGCTACGGTCCAATGAGCTAGCCAGTT**G**TGTGG
ATATGGCATATGATTGTGTTGACGAGTGCGGCTCCGAGCTTG**TC**G**G....**TATGCCC**G**
TGGCGGGGGCTCTTGTCAACGAAAACAAT**G**AATTGGGCAT**..**GCTGGTTAAACT**G**AA
CACTGCCTT**G**GTAGCACCACGTAGGTTTCT**G**GAGTAATATAATGTTTGTTT

***Hv*PEI5**

TCTAGGTAGCCTTGCACCAATGACAACAACTACAACCTCTATATTCTTCTCCGCCAT
TCTATT**(T/C)**AGTCTTCTTTCTGTTGCAATCACTGGTGAGAC**(A/C)**A**(T/A)**TGAGTCTG
GCAGTGGTGGGGCCCGGGTGACGAACCTCATTGTGGAAGCATGCAAGAACGCAT
CAGGCTATCGTCGTGGTGTCACAAATTTCACACAGGAATTTTGCTTGTCAACCCTT
CAGTCGGACAATAGGACTGTGGAGGCAAAGGATCACCTTGAACTGGTGGTCATTG
CCATCGACATCCTTAAAGGCCGCCTCACTACTGCTAATCACAACATTGATAAAATG
CTACAAAATGCAAAGAAAGGCACAGTGCCAATGCGCGATCTCAGTTGCTGCAAGG
TGTACTATGATACAACAATGAGAATCATCAATATATGTGATTACATGATCACAGACT
TCCG**(C/T)**GGACATAAGGGCAGGCTGAAGTCCTTGGAGCTTCCTCGTTGTGTTGAC
AGAGCAGGCTACCCAGTCGACGACTGCTGGTCCGATCTTGAGTACAATATGCCAT
GGGCCGATGCACTCATCAGAGAAAATCTCGAGATTGCCGTGCTGGTCAGCCTCGA
CTATGCCTTGCTAGCACCATATGATGTCAGTGATTAATATAATGTTAGTTTTATCTGT
AATAAATGAT

>Atlas (Haplotype H1)

ATGACAACAACTACAACCTCTATATTCTTCTCCGCCATTCTATT**T**AGTCTTCTTTCTG
TTGCAATCACTGGTGAGAC**A**A**T**TGAGTCTGGCAGTGGTGGGGCCCGGGTGACGAA
CCTCATTGTGGAAGCATGCAAGAACGCATCAGGCTATCGTCGTGGTGTCACAAATT
TCACACAGGAATTTTGCTTGTCAACCCTTCAGTCGGACAATAGGACTGTGGAGGCA
AAGGATCACCTTGAACTGGTGGTCATTGCCATCGACATCCTTAAAGGCCGCCTCAC
TACTGCTAATCACAACATTGATAAAATGCTACAAAATGCAAAGAAAGGCACAGTGC
CAATGCGCGATCTCAGTTGCTGCAAGGTGTACTATGATACAACAATGAGAATCATC
AATATATGTGATTACATGATCACAGACTTCCG**C**GGACATAAGGGCAGGCTGAAGTC
CTTGGAGCTTCCTCGTTGTGTTGACAGAGCAGGCTACCCAGTCGACGACTGCTGG
TCCGATCTTGAGTACAATATGCCATGGGCCGATGCACTCATCAGAGAAAATCTCGA
GATTGCCGTGCTGGTCAGCCTCGACTATGCCTTGCTAGCACCATATGATGTCAGTG
ATTAATATAATGTTAGTTTTATCTGTAATAAATGAT

>Quinn (Haplotype H2)

ATGACAACAACTACAACCTCTATATTCTTCTCCGCCATTCTATT**C**AGTCTTCTTTCTG
TTGCAATCACTGGTGAGAC**C**A**A**TGAGTCTGGCAGTGGTGGGGCCCGGGTGACGA

ACCTCATTGTGGAAGCATGCAAGAACGCATCAGGCTATCGTCGTGGTGTCACAAAT
TTCACACAGGAATTTTGCTTGTCAACCCTTCAGTCGGACAATAGGACTGTGGAGGC
AAAGGATCACCTTGAACTGGTGGTCATTGCCATCGACATCCTTAAAGGCCGCCTCA
CTACTGCTAATCACAACATTGATAAAATGCTACAAAATGCAAAGAAAGGCACAGTG
CCAATGCGCGATCTCAGTTGCTGCAAGGTGTACTATGATACAACAATGAGAATCAT
CAATATATGTGATTACATGATCACAGACTTCCGTGGACATAAGGGCAGGCTGAAGT
CCTTGGAGCTTCCTCGTTGTGTTGACAGAGCAGGCTACCCAGTCGACGACTGCTG
GTCCGATCTTGAGTACAATATGCCATGGGCCGATGCACTCATCAGAGAAAATCTCG
AGATTGCCGTGCTGGTCAGCCTCGACTATGCCTTGCTAGCACCATATGATGTCAGT
GATTAATATAATGTTAGTTTTATCTGTAATAAATGAT

***Hv*PEI6**

TTTATCTCCACCTTCGCCAACTTCGA(T/C)A(C/T)ATTTCAAGACTTCCAACTAGGCT
TATAGCAACG(G/A/.)CAACAATGTCGGTGACCT(T/C)CATCCT(T/C)TTCTCTGCCAT
CACTATCATGCTCCTTTC(C/T)ACTACCATCGC(T/A)(G/A)CTGAATCTACTGCCTCT
GGTGGTGGAAACCCCAAGGC(A/G)ACCAACTTCATGGTGGAAGC(C/G)TGCAAGAA
TGC(G/A)TCAACGAAAA(A/G)CCAAATATACGACCCCAACCCAATC(G/A)CACAGGA
GTT(T/C)TGTGTGT(C/T)GA(C/G)CCTCAAGTTGGACAATCGGAGCGCCGAGGCTAA
AGATGTCCATAGCCTGGTACACGTCGCCATT(G/C)ATATACTCAAGGGACAAGTTG
CCACGGCCAATGACAATGTCAAACAAATG(C/A)TGCAC(A/G)ACACCAA(G/A)AATG
GCACGTCTACAATGCGTTCCCTCAGTTTCTGC(A/G)TGGT(G/C)GA(C/T)TACAACCG
CATGGTGA(G/C)CATCCTCA(A/G)CATATGTGACACAATGATAAATGAGTAC
(A/C)ACGGTCGCA(A/C)GGGCAGAGCCGA(T/C)GA(C/T)GGGTTGCTATCTTCTGAG
CTGCCCGCCTGTGTGGAGAAAGTTGACAAACCTTTCATCGACTG(T/C)TGGTTGGG
CCTTCTTGGTATGGAGG(C/T)(G/C)AAAAAGCTATTAGA(T/C)GA(G/A)AAC(T/A)TT(G
/T)CGGTGGGCATGTTGGTCAAA(C/G)TCAACTTTT(G/A)(C/T)CTTGCTAGCATCATA
TAGGTTTAATGAT

>Atlas (Haplotype H1)

TTTATCTCCACCTTCGCCAACTTCGATACATTTCAAGACTTCCAACTAGGCTTATAG
CAACGGCAACAATGTCGGTGACCTTCATCCTTTTCTCTGCCATCACTATCATGCTC
CTTTCCACTACCATCGCTGCTGAATCTACTGCCTCTGGTGGTGGAAACCCCAAGGC
AACCAACTTCATGGTGGAAGCCTGCAAGAATGCGTCAACGAAAAACCAAATATACG
ACCCCAACCCAATCGCACAGGAGTTTTGTGTGTCGACCCTCAAGTTGGACAATCG
GAGCGCCGAGGCTAAAGATGTCCATAGCCTGGTACACGTCGCCATTGATATACTC
AAGGGACAAGTTGCCACGGCCAATGACAATGTCAAACAAATGCTGCACAACACCA
AGAATGGCACGTCTACAATGCGTTCCCTCAGTTTCTGCATGGTGGACTACAACCGC
ATGGTGAGCATCCTCAACATATGTGACACAATGATAAATGAGTACAACGGTCGCAA
GGGCAGAGCCGATGACGGGTTGCTATCTTCTGAGCTGCCCGCCTGTGTGGAGAAA
GTTGACAAACCTTTCATCGACTGTTGGTTGGGCCTTCTTGGTATGGAGGCGAAAAA
GCTATTAGATGAGAACTTTGCGGTGGGCATGTTGGTCAAACTCAACTTTTGCCTTG
CTAGCATCATATAGGTTTAATGAT

> Abyssinian (Haplotype H2)

TTTATCTCCACCTTCGCCAACTTCGATACATTTCAAGACTTCCAACTAGGCTTATAG
CAACGGCAACAATGTCGGTGACCTCCATCCTCTTCTCTGCCATCACTATCATGCTC
CTTTCCACTACCATCGCTGCTGAATCTACTGCCTCTGGTGGTGGAAACCCCAAGGC
AACCAACTTCATGGTGGAAGCCTGCAAGAATGCGTCAACGAAAAACCAAATATACG
ACCCCAACCCAATCACACAGGAGTTCTGTGTGTCGACCCTCAAGTTGGACAATCG
GAGCGCCGAGGCTAAAGATGTCCATAGCCTGGTACACGTCGCCATTGATATACTC
AAGGGACAAGTTGCCACGGCCAATGACAATGTCAAACAAATGCTGCACAACACCA
AGAATGGCACGTCTACAATGCGTTCCCTCAGTTTCTGCATGGTGGACTACAACCGC
ATGGTGAGCATCCTCAACATATGTGACACAATGATAAATGAGTACAACGGTCGCAC
GGGCAGAGCCGATGATGGGTTGCTATCTTCTGAGCTGCCCGCCTGTGTGGAGAAA
GTTGACAAACCTTTCATCGACTGCTGGTTGGGCCTTCTTGGTATGGAGGCGAAAAA
GCTATTAGATGAGAACTTTTCGGTGGGCATGTTGGTCAAACTCAACTTTTGCCTTG
CTAGCATCATATAGGTTTAATGAT

> Alexis (Haplotype H3)

TTTATCTCCACCTTCGCCAACTTCGATACATTTCAAGACTTCCAACTAGGCTTATAG
CAACGACAACAATGTCGGTGACCTCCATCCTCTTCTCTGCCATCACTATCATGCTC
CTTTCCACTACCATCGCTGCTGAATCTACTGCCTCTGGTGGTGGAAACCCCAAGGC
AACCAACTTCATGGTGGAAGCCTGCAAGAATGCGTCAACGAAAAACCAAATATACG
ACCCCAACCCAATCACACAGGAGTTCTGTGTGTCGACCCTCAAGTTGGACAATCG
GAGCGCCGAGGCTAAAGATGTCCATAGCCTGGTACACGTCGCCATTGATATACTC
AAGGGACAAGTTGCCACGGCCAATGACAATGTCAAACAAATGCTGCACAACACCA
AGAATGGCACGTCTACAATGCGTTCCCTCAGTTTCTGCATGGTGGACTACAACCGC
ATGGTGAGCATCCTCAACATATGTGACACAATGATAAATGAGTACAACGGTCGCAC
GGGCAGAGCCGATGATGGGTTGCTATCTTCTGAGCTGCCCGCCTGTGTGGAGAAA
GTTGACAAACCTTTCATCGACTGCTGGTTGGGCCTTCTTGGTATGGAGGCGAAAAA
GCTATTAGATGAGAACTTTTCGGTGGGCATGTTGGTCAAAGTCAACTTTTGCCTTG
CTAGCATCATATAGGTTTAATGAT

> Morex (Haplotype H4)

TTTATCTCCACCTTCGCCAACTTCGACACATTTCAAGACTTCCAACTAGGCTTATAG
CAACGGCAACAATGTCGGTGACCTCCATCCTCTTCTCTGCCATCACTATCATGCTC
CTTTCCACTACCATCGCAACTGAATCTACTGCCTCTGGTGGTGGAAACCCCAAGGC
GACCAACTTCATGGTGGAAGCGTGCAAGAATGCGTCAACGAAAAGCCAAATATAC
GACCCCAACCCAATCACACAGGAGTTTTGTGTGTTGAGCCTCAAGTTGGACAATCG
GAGCGCCGAGGCTAAAGATGTCCATAGCCTGGTACACGTCGCCATTGATATACTC
AAGGGACAAGTTGCCACGGCCAATGACAATGTCAAACAAATGATGCACGACACCA
AAAATGGCACGTCTACAATGCGTTCCCTCAGTTTCTGCGTGGTCGATTACAACCGC
ATGGTGAGCATCCTCAGCATATGTGACACAATGATAAATGAGTACCACGGTCGCAC
GGGCAGAGCCGATGATGGGTTGCTATCTTCTGAGCTGCCCGCCTGTGTGGAGAAA
GTTGACAAACCTTTCATCGACTGTTGGTTGGGCCTTCTTGGTATGGAGGTGAAAAA
GCTATTAGATGAAAACATTGCGGTGGGCATGTTGGTCAAACTCAACTTTTATCTTG
CTAGCATCATATAGGTTTAATGAT

> Opal (Haplotype H5)

TTTATCTCCACCTTCGCCAACTTCGATACATTTCAAGACTTCCAACTAGGCTTATAG
CAACGGCAACAATGTCGGTGACCTCCATCCTCTTCTCTGCCATCACTATCATGCTC
CTTTCTACTACCATCGCTGCTGAATCTACTGCCTCTGGTGGTGGAAACCCCAAGGC
AACCAACTTCATGGTGGAAGCCTGCAAGAATGCGTCAACGAAAAACCAAATATACG
ACCCCAACCCAATCACACAGGAGTTTTGTGTGTCGACCCTCAAGTTGGACAATCG
GAGCGCCGAGGCTAAAGATGTCCATAGCCTGGTACACGTCGCCATTGATATACTC
AAGGGACAAGTTGCCACGGCCAATGACAATGTCAAACAAATGCTGCACAACACCA
AGAATGGCACGTCTACAATGCGTTCCCTCAGTTTCTGCATGGTGGACTACAACCGC
ATGGTGAGCATCCTCAACATATGTGACACAATGATAAATGAGTACAACGGTCGCAA
GGGCAGAGCCGATGATGGGTTGCTATCTTCTGAGCTGCCCGCCTGTGTGGAGAAA
GTTGACAAACCTTTCATCGACTGTTGGTTGGGCCTTCTTGGTATGGAGGCGAAAAA
GCTATTAGATGAGAACTTTGCGGTGGGCATGTTGGTCAAACTCAACTTTTGCCTTG
CTAGCATCATATAGGTTTAATGAT

> Golden Promise (Haplotype H6)

TTTATCTCCACCTTCGCCAACTTCGATACATTTCAAGACTTCCAACTAGGCTTATAG
CAACG.CAACAATGTCGGTGACCTCCATCCTCTTCTCTGCCATCACTATCATGCTCC
TTTCCACTACCATCGCTGCTGAATCTACTGCCTCTGGTGGTGGAAACCCCAAGGCA
ACCAACTTCATGGTGGAAGCCTGCAAGAATGCATCAACGAAAAACCAAATATACGA
CCCCAACCCAATCACACAGGAGTTTTGTGTGTCGACCCTCAAGTTGGACAATCGG
AGCGCCGAGGCTAAAGATGTCCATAGCCTGGTACACGTCGCCATTGATATACTCAA
GGGACAAGTTGCCACGGCCAATGACAATGTCAAACAAATGCTGCACAACACCAAG
AATGGCACGTCTACAATGCGTTCCCTCAGTTTCTGCATGGTGGACTACAACCGCAT
GGTGAGCATCCTCAACATATGTGACACAATGATAAATGAGTACAACGGTCGCAAG
GGCAGAGCCGACGATGGGTTGCTATCTTCTGAGCTGCCCGCCTGTGTGGAGAAAG
TTGACAAACCTTTCATCGACTGTTGGTTGGGCCTTCTTGGTATGGAGGCGAAAAAG
CTATTAGACGAGAACTTTGCGGTGGGCATGTTGGTCAAACTCAACTTTTGCCTTGC
TAGCATCATATAGGTTTAATGAT

> Cebada Atlas (Haplotype H7)

TTTATCTCCACCTTCGCCAACTTCGATACATTTCAAGACTTCCAACTAGGCTTATAG
CAACGGCAACAATGTCGGTGACCTTCATCCTTTTCTCTGCCATCACTATCATGCTC
CTTTCCACTACCATCGCTGCTGAATCTACTGCCTCTGGTGGTGGAAACCCCAAGGC
AACCAACTTCATGGTGGAAGCCTGCAAGAATGCGTCAACGAAAAACCAAATATACG
ACCCCAACCCAATCACACAGGAGTTTTGTGTGTCGACCCTCAAGTTGGACAATCG
GAGCGCCGAGGCTAAAGATGTCCATAGCCTGGTACACGTCGCCATTGATATACTC
AAGGGACAAGTTGCCACGGCCAATGACAATGTCAAACAAATGCTGCACAACACCA
AGAATGGCACGTCTACAATGCGTTCCCTCAGTTTCTGCATGGTGGACTACAACCGC
ATGGTGACCATCCTCAACATATGTGACACAATGATAAATGAGTACAACGGTCGCAA
GGGCAGAGCCGATGACGGGTTGCTATCTTCTGAGCTGCCCGCCTGTGTGGAGAAA
GTTGACAAACCTTTCATCGACTGTTGGTTGGGCCTTCTTGGTATGGAGGCGAAAAA
GCTATTAGATGAGAACTTTGCGGTGGGCATGTTGGTCAAACTCAACTTTTGCCTTG
CTAGCATCATATAGGTTTAATGAT

> Turk 8886 (Haplotype H8)

TTTATCTCCACCTTCGCCAACTTCGA**T**A**T**ATTTCAAGACTTCCAACTAGGCTTATAG
CAACG**G**CAACA<u>ATG</u>TCGGTGACCT**C**CATCCT**C**TTCTCTGCCATCACTATCATGCTC
CTTTC**C**ACTACCATCGC**TG**CTGAATCTACTGCCTCTGGTGGTGGAAACCCCAAGGC
AACCAACTTCATGGTGGAAGC**C**TGCAAGAATGC**G**TCAACGAAAA**A**CCAAATATACG
ACCCCAACCCAATC**A**CACAGGAGTT**C**TGTGTGT**C**GA**C**CCTCAAGTTGGACAATCG
GAGCGCCGAGGCTAAAGATGTCCATAGCCTGGTACACGTCGCCATT**C**ATATACTC
AAGGGACAAGTTGCCACGGCCAATGACAATGTCAAACAAATG**C**TGCAC**A**ACACCA
A**G**AATGGCACGTCTACAATGCGTTCCCTCAGTTTCTGC**A**TGGT**G**GA**C**TACAACCGC
ATGGTGA**G**CATCCTCA**A**CATATGTGACACAATGATAAATGAGTAC**A**ACGGTCGCA**C**
GGGCAGAGCCGA**T**GA**T**GGGTTGCTATCTTCTGAGCTGCCCGCCTGTGTGGAGAAA
GTTGACAAACCTTTCATCGACTG**C**TGGTTGGGCCTTCTTGGTATGGAGG**CC**AAAAA
GCTATTAGA**T**GA**G**AAC**T**TT**T**CGGTGGGCATGTTGGTCAAA**C**TCAACTTTT**GC**CTTG
CTAGCATCATA<u>TAG</u>GTTTAATGAT
