# Supplementary material for: Are PECTIN ESTERASE INHIBITOR Genes Involved in Mediating Resistance to Rhynchosporium commune in Barley?
Source: PLoS One. 2016 Mar 3;11(3):e0150485. doi: 10.1371/journal.pone.0150485 (PMC4777559; doi:10.1371/journal.pone.0150485)
Supplement: S2 Table — (PDF) [file pone.0150485.s006.pdf]

PLOS ONE Supporting Information

Article title: **Are PECTIN ESTERASE INHIBITOR genes involved in mediating resistance to Rhynchosporium commune in barley?**

Authors: Stephan Marzin, Anja Hanemann, Shailendra Sharma, Götz Hensel, Jochen Kumlehn, Günther Schweizer, Marion S. Röder

The following Supporting Information is available for this article:

Table S2: Primer for amplification of full-length sequences of 6 PEI genes in barley.

Gene	Primer	Fragment	Sequence
HvPEI1	PEIC-1L<br>PEIC-1R	765 bp	ATCAAAGCGCACATCCATTT<br>CATACACCACTCAGGCTAAAATACATA
HvPEI2	PEI2-1L<br>PEI2-1R	777 bp	GTCTAACAATTCATCCCCACCTCT<br>ACAACATTATTATTGTTTTTGCAAACTG
HvPEI3	FL-PEI3_F2<br>FL-PEI3_R2	742 bp	GCAAGCCTAGCAGCAATGAAAACG<br>TTACATTATATCTTTATTTGCTTTCATACCCCA
HvPEI4	FL-PEI4_F1<br>FL-PEI4_R1	816 bp	CATTCTATATCCATTTATCCACACCTCTCTA<br>GAAGGAGTAGTACAATTGCTGCCG
HvPEI4	FL-PEI4_F3<br>FL-PEI4_R3	1188 bp	GTTGCATTAATGAACATGTTGAAGT<br>CGCGGACGGGCCCAAAAC
HvPEI5	FL-PEI5_F<br>FL-PEI5_R	931 bp	AAGATCCCAAATTGAGCATACTACA<br>CTGCATCATTATTCGAAAGAGTGT
HvPEI6	3681-82_F<br>3681-82_R	780 bp	CCTACCCAGCGTTCTATATCCATTT<br>CAATCCAAGTGAAGGTAAACAGA
