# Supplementary material for: Are PECTIN ESTERASE INHIBITOR Genes Involved in Mediating Resistance to Rhynchosporium commune in Barley?
Source: PLoS One. 2016 Mar 3;11(3):e0150485. doi: 10.1371/journal.pone.0150485 (PMC4777559; doi:10.1371/journal.pone.0150485)
Supplement: S3 Table — (PDF) [file pone.0150485.s007.pdf]

PLOS ONE Supporting Information

Article title: Are PECTIN ESTERASE INHIBITOR genes involved in mediating resistance to Rhynchosporium commune in barley?

Authors: Stephan Marzin, Anja Hanemann, Shailendra Sharma, Götz Hensel, Jochen Kumlehn, Günther Schweizer, Marion S. Röder

The following Supporting Information is available for this article:

Table S3: Primer for assessment of transcript abundance and transgene copy number determination.

	Name	Orientation	5´ Label	Sequence (5´- 3´)	3´Quencher	Length (bp)
**Expression**	qRT2_PEI2_F	forward primer		CATCGCCGTCGACATCCTCAAG		22
	qRT2_PEI2_R	reverse primer		AGCATGGCGTCGCAGAGGC		19
	qRT_PEI3_F7	forward primer		GCTTCACGCATGACTTCTGTGTATCG		26
	qRT_PEI3_R7	reverse primer		GCGCGAGTCGCAGTCTTGTCC		21
	qRT_PEI4_Fa	forward primer		CGAAGCATGGAGGCAAAGGACC		22
	qRT_PEI4_R	reverse primer		CTGAGGACACGCATTGGCACTG		22
**Copy Number**	TM_Hpt_F	forward primer		GCGAAGAATCTCGTGCTTTC		20
	TM_Hpt_R	reverse primer		ATAGGTCAGGCTCTCGCTGA		20
	TM_Hpt_Sonde		FAM	AATAGCTGCGCCGATGGTTT	BHQ1	20
**Normalization**	HvUBC_F	forward primer		AAGCAGCCAGAATGTACAGCGAGAAC		26
	HvUBC_R	reverse primer		GGTACAGACCAGCAAAGCCAGAAATG		26
	TM_HvUBC_F	forward primer		ACTCCGAAGCAGCCAGAATG		20
	TM_HvUBC_R	reverse primer		GATCAAGCACAGGGACACAAC		21
	TM_HvUBC_Sonde		JOE	GAGAACAAGCGCGAGTACAACCGCAAGGTG	BHQ1	30
